# Diversity of symptom phenotypes in SARS-CoV-2 community infections observed in multiple large datasets

**DOI:** 10.1038/s41598-023-47488-9

**Published:** 2023-12-07

**Authors:** Martyn Fyles, Karina-Doris Vihta, Carole H Sudre, Harry Long, Rajenki Das, Caroline Jay, Tom Wingfield, Fergus Cumming, William Green, Pantelis Hadjipantelis, Joni Kirk, Claire J Steves, Sebastien Ourselin, Graham F Medley, Elizabeth Fearon, Thomas House

**Affiliations:** 1 Department of Mathematics, University of Manchester, Manchester, UK; 2 The Alan Turing Institute for Data Science and Artificial Intelligence, London, NW1 2DB UK; 3 United Kingdom Health Security Agency (UKHSA), London, UK; 4 Nuffield Department of Medicine, University of Oxford, Oxford, UK; 5 Department of Engineering, University of Oxford, Oxford, UK; 6 National Institute for Health Research Health Protection Research Unit in Healthcare Associated Infections and Antimicrobial Resistance, University of Oxford, Oxford, UK; 7 School of Biomedical Engineering & Imaging Sciences, King’s College London, London, UK; 8 MRC Unit for Lifelong Health and Ageing, University College London, London, UK; 9 Department of Computer Science, University of Manchester, Oxford Road, Manchester, M13 9PL UK; 10 Department of Clinical Sciences and International Public Health, Liverpool School of Tropical Medicine, Liverpool, L3 5QA UK; 11 Tropical and Infectious Disease Unit, Liverpool University Hospitals NHS Foundation Trust, Liverpool, L7 8XP UK; 12 WHO Collaborating Centre on Tuberculosis and Social Medicine, Department of Global Public Health, Karolinska Institutet, 171 77 Stockholm, Sweden; 13 Department of Twin Research and Genetic Epidemiology King’s College London, London, UK; 14 Department of Ageing and Health Guy’s and St Thomas’ NHS Foundation Trust, London, UK; 15 Centre for the Mathematical Modelling of Infectious Disease, London School of Hygiene and Tropical Medicine, London, WC1E 7HT UK; 16 Department of Global Health and Development, London School of Hygiene and Tropical Medicine, London, WC1E 7HT UK; 17 Institute for Global Health, University College London, London, UK; 18 IBM Research, Hartree Centre, Daresbury, WA4 4AD UK

**Keywords:** Statistics, Viral infection, Respiratory signs and symptoms

## Abstract

Variability in case severity and in the range of symptoms experienced has been apparent from the earliest months of the COVID-19 pandemic. From a clinical perspective, symptom variability might indicate various routes/mechanisms by which infection leads to disease, with different routes requiring potentially different treatment approaches. For public health and control of transmission, symptoms in community cases were the prompt upon which action such as PCR testing and isolation was taken. However, interpreting symptoms presents challenges, for instance, in balancing the sensitivity and specificity of individual symptoms with the need to maximise case finding, whilst managing demand for limited resources such as testing. For both clinical and transmission control reasons, we require an approach that allows for the possibility of distinct symptom phenotypes, rather than assuming variability along a single dimension. Here we address this problem by bringing together four large and diverse datasets deriving from routine testing, a population-representative household survey and participatory smartphone surveillance in the United Kingdom. Through the use of cutting-edge unsupervised classification techniques from statistics and machine learning, we characterise symptom phenotypes among symptomatic SARS-CoV-2 PCR-positive community cases. We first analyse each dataset in isolation and across age bands, before using methods that allow us to compare multiple datasets. While we observe separation due to the total number of symptoms experienced by cases, we also see a separation of symptoms into gastrointestinal, respiratory and other types, and different symptom co-occurrence patterns at the extremes of age. In this way, we are able to demonstrate the deep structure of symptoms of COVID-19 without usual biases due to study design. This is expected to have implications for the identification and management of community SARS-CoV-2 cases and could be further applied to symptom-based management of other diseases and syndromes.

## Introduction

Since the identification of the SARS-CoV-2 virus, the COVID-19 pandemic has led to over 700 million confirmed cases and 7 million confirmed deaths. In response to this, one of the largest ever public health responses has been mounted, with over 13 billion vaccine doses administered and a large variety of non-pharmaceutical interventions that have fundamentally changed behaviour and healthcare provision around the world since the start of 2020^1–3^.

Understanding the clinical presentation and course of SARS-CoV-2 infection has been central for transmission control, particularly in determining policy for identification of cases for isolation and tracing of their contacts^4,5^, and for prediction of clinical outcomes. COVID-19 cases can present with symptoms from a wide range of categories: respiratory, systemic, cardiovascular and gastrointestinal^6^, with high variability in severity of disease between individuals depending strongly on age, and on factors including comorbidities^7,8^. In addition, a significant proportion of infections are estimated to remain asymptomatic^9^. Analyses of symptom clustering patterns amongst hospitalised patients have helped to improve clinical care^10^, while longitudinal clustering approaches contribute to early identification of cases more likely to experience severe outcomes^11^, though these cases will be those with the more severe disease course.

Targeted population transmission control policies that do not simply require the whole population to avoid contact, with all the damage that this entails, require identifying infectious cases. In the absence of regular population-wide screening approaches, case identification requires a symptoms-based approach, and this was a central pillar in the UK’s COVID-19 response from May 2020 until April 2022. Over this time period, PCR testing was initiated when individuals from the broader population experienced at least one of; fever, new continuous cough or loss of taste or smell. Because PCR testing requires laboratories and associated staffing and logistical networks, there was a need to balance the sensitivity of symptom criteria for testing with specificity, given the varieties of infections and syndromes that could give rise to the relevant symptoms. Thus, the effectiveness of testing, contact tracing and isolation policies were dependent in part on the performance of the symptom criteria for testing. A number of single study analyses in the UK have sought to investigate and to improve upon these criteria, sequentially adding or dropping symptoms to better optimise the sensitivity and specificity trade-off^12,13^. While valuable, these approaches essentially assume a single phenotype, whereas it is possible that multiple phenotypes exist and, therefore, that the trade-off cannot be optimised in this manner.

Assessment of the diversity of genotypes and (endo-)phenotypes is a long-standing tool in both infectious diseases and chronic non-communicable diseases, which has been significantly accelerated by modern experimental and theoretical techniques^14–16^. In particular, such analysis often helps with the standard process of identifying multiple disease aetiologies with the same presentation, or vice versa, a single disease with highly variable outcomes. This latter distinction is particularly important for COVID-19, where different courses of action, including public health interventions, are taken depending on symptom status^17^. Beyond the acute phase of the disease, consideration of different potential ‘long COVID’ phenotypes based on symptom status could help to identify more or less appropriate treatment approaches.

Here, to investigate the presence of distinct COVID-19 symptom phenotypes, we investigate patterns of symptom *occurrence*, *co-occurrence* and *clustering* in PCR-positive symptomatic SARS-CoV-2 cases – previously considered predominantly in hospitalisation data heavily skewed towards more severe infections^10,11,18^ – in four very large community-based datasets.

## Methods

### Data

#### Population and setting

We examine identified infections for the time period May 2020 to March 2021 in the UK. Due to this data collection time period, and the effects of vaccination on preventing disease, we expect the datasets to contain predominantly unvaccinated individuals. These datasets are diverse in their sampling and data collection methods and include (a) 1,637,965 symptomatic cases from ‘Pillar 2’ testing data from the National Health Service (NHS) Test and Trace system, designed to capture cases in the general population; (b) 112,925 symptomatic cases from the Second Generation Surveillance System (SGSS) in England’s national laboratory reporting system, which includes cases associated with healthcare settings among patients and healthcare staff; (c) 52,084 symptomatic self-reported cases from the COVID-19 Symptom Study (CSS), which uses a smartphone app associated with https://COVID.joinzoe.com/ to collect daily symptom reports; and (d) 9,166 symptomatic cases from The Office for National Statistics COVID-19 Infection Survey (CIS), a longitudinal study of a representative sample of UK households.

#### NHS test and trace routine testing data

NHS Test and Trace data is further split into two parts: Pillar 2, cases detected in the community, usually on the basis of symptoms to initiate testing; and the Second Generation Surveillance System (SGSS), for people tested in healthcare settings. In May 2020, the UK government made PCR testing available for individuals who had one of the following symptoms: a new, continuous cough; fever; loss of taste or loss of smell. These tests are reported through Pillar 2, through which several different avenues to testing are available. Individuals can book a test appointment through a government website for either a drive-in or walk-through testing centre, where they self-swab their nose and throat (under some supervision, with an adult carer conducting the swabbing for children), with the swab then sent to a lab for PCR testing. Alternatively, individuals can order home test kits where they self-swab at home and post the kit back, with the swab again sent to a lab for PCR testing. If the individual tests positive, their case is transferred to NHS Test and Trace who contact cases to inform them of their result and ask them to conduct a questionnaire including symptoms experienced. The questionnaire is conducted either via a web form or over the phone with a trained contact tracer. Since the end of 2020, Pillar 2 has also included positive cases identified using rapid antigen tests among people not experiencing one of the PCR test prompting symptoms. These tests also use a nasopharyngeal swab and are conducted at the home, workplace or school and, if positive, are requested to be followed up by a confirmatory PCR test (though policy has varied over time). Reported positive cases from asymptomatic testing are also followed up by NHS Test and Trace.

The Second Generation Surveillance System (SGSS) dataset includes people who test because they work in or have been tested in a healthcare setting as a patient. This latter group includes both those in hospital because of severe COVID-19 symptoms, but also those in hospital for other reasons but receiving SARS-CoV-2 testing. Thus they are likely, to be more severe cases in the SGSS versus Pillar 2 data, but not exclusively. Again, individuals are swabbed and PCR tested, with their case transferred to NHS Test and Trace if testing positive for symptom reporting and contact tracing.

#### COVID symptom study (CSS)

The CSS is a participatory surveillance study collecting data via a smartphone app. It is led by Kings College London and Zoe Global Ltd and was initiated in March 2020 in the United Kingdom and the United States^19^. Individuals are asked to report daily whether they are feeling ‘physically normal’ that day and, if not, what symptoms they are experiencing. As well as demographic data that is collected upon sign-up, participants are also asked to self-report whether they have had any tests for SARS-CoV-2 infection and, if so, the date of the test and its result. Demographic data and data about underlying conditions are collected at first registration. Participants can also proxy-report for children or for others they care for (e.g elderly adults they care for).

As well as enabling individuals to self-report COVID-19 testing that they have undertaken via the UK’s routine testing programmes or surveillance studies, the CSS invites individuals to complete a PCR test via routine testing if they 1) have made at least one report of no symptoms in the previous week and 2) report a new symptom not on the list to prompt symptomatic testing (e.g sore throat). This means that we might expect the CSS reporting to be less dominated by the symptoms required to initiate symptom-based testing than the Pillar 2 routine testing dataset.

#### ONS COVID-19 infection survey (CIS)

The CIS is a UK population-representative survey of households randomly selected continuously since April 2020 from address lists and previous surveys^20^. Households are followed longitudinally with weekly visits for the first month and monthly visits for 12 months from enrolment. A fieldworker attends enrolled households each visit for testing for household members aged 2 years and above and to conduct an interview including, among other topics, demographic data (reported at the first visit) and symptoms experienced over the previous 7 days. At each visit, participants conduct a nose and throat swab under the supervision of a fieldworker. These swabs are sent for PCR testing, and the result is communicated to participants. At the same time as swabbing, all participants are also interviewed by the fieldworker to complete a symptom questionnaire.

### Data extraction and preparation

From each dataset, we extract all PCR-positive individuals and associate them with symptoms experienced within a time window of the test appropriate for the dataset. More detail about each dataset, data collection and extraction are given in the Supplementary Materials. For the *i*-th individual and *a*-th symptom, we let $X_{ia} = 1$ if the symptom is present during the time window around the positive test and $X_{ia} = 0$ otherwise. For a dataset with *n* individuals measuring *p* symptoms, we can then construct an $n\times p$ matrix $[X_{ia}]$, where the rows of this matrix form a set of *n* length-*p* feature vectors for individuals, $\{\textbf{y}_i \}$, and the columns form a set of *p* length-*n* feature vectors for symptoms, $\{\textbf{x}_a \}$, each of which can then be used as input for unsupervised learning algorithms. In addition to descriptive analysis of the data, we used three complementary approaches to looking at clustering and co-occurrence of symptoms.

#### Sample populations

The dates over which cases are collected from each study are shown in Table 1 and in total cover the period from April 2020 to March 2021, with the largest overlap between November 2020 and January 2021. We make no exclusions based on age or other characteristics.

**Table 1:** Descriptive statistics of the population in each dataset.

Variable	Dataset			
	Pillar 2	SGSS	CSS	CIS
*Data collection variables*				
Start date	29/11/2020	29/11/2020	11/05/2020	28/04/2020
End Data	28/03/2021	28/03/2021	11/01/2021	13/03/2021
Location of participants	England	England	UK	UK
*Age variables*				
Mean (years)	38	48	-	43
Median (years)	37	47	40-49	45
IQR (years)	26–51	32–62	–	29–57
*Sex breakdown*				
Male	736,906 (45.0%)	42,355 (40.3%)	23,540 (38.2%)	4,142 (45.2%)
Female	875,545 (55.0%)	62,808 (59.7%)	38,051 (61.8%)	5,024 (54.8%)
Intersex	–	–	3	–
N/A	–	–	29	–
*Sample sizes*				
Total dataset size	1,898,273	179,550	61,623	27,903
Symptomatic cases	1,637,965 (86.3%)	112,925 (62.9%)	52,084 (84.5%)	9,166 (32.8%)
Asymptomatic cases	260,308 (13.7%)	66,625 (37.1%)	9,539 (15.5%)	18,737 (67.2%)

From each dataset, we include only cases that report at least one symptom within the symptom reporting window around a positive test (detailed below and listed in Table 2). For NHS Test and Trace data, positive cases who are never reached and interviewed post-testing are not included in this dataset. The definition of ‘symptomatic’ necessarily varies across the datasets because there are differences in the full list of symptoms asked about. Symptoms that were not core dataset variables and were instead recorded by manual entry were not included. For each dataset, we chose to include all dataset symptoms from each study (except for ‘write-in’ symptoms), rather than excluding symptoms that were not common across all. This was with the intention of maximising the amount of symptom information available for analysis. We also extracted demographic information.

**Table 2:** Symptom survey questions by dataset.

Variable	Dataset		
	Test and Trace (Pillar 2 & SGSS)	CIS	CSS
Symptomatic	Are you experiencing any of the following symptoms? Please select at least one. (cases may select “I have no symptoms”)	Have you had any of the following symptoms in the last 7 days?	Are you feeling physically normal today? (I feel physically normal; I do not feel physically normal)
Abdominal pain	–	Abdominal pain	Do you have an unusual abdominal pain?
Altered consciousness	Altered consciousness	–	–
Altered/loss of smell	–	–	Do you have a loss of smell/taste?
Chest pain	–	–	Are you feeling an unusual chest pain or tightness in your chest?
Cough	A new, continuous cough	Cough	Do you have a persistent cough?
Delirium	–	–	Do you have any of the following symptoms: confusion, disorientation, or drowsiness?
Diarrhoea	Diarrhoea	Diarrhoea	Are you experiencing diarrhoea?
Fatigue	Extreme tiredness	Weakness/tiredness	Are you experiencing unusual fatigue? (mild; severe)*
Fever	High temperature or fever (higher than 38$^{\circ }$C)	Fever	Do you have a fever?
Headache	Headache	Headache	Do you have a headache?

Hoarse voice	–	–	Do you have an unusually hoarse voice?
Joint pain	Joint pain	–	–
Loss of appetite	Loss of appetite	–	Have you been skipping meals?
Loss of smell	–	Loss of smell	–
Loss of smell or taste	Loss or change to your sense of smell or taste (you cannot smell or taste anything, or things smell or taste different to normal)	–	–
Loss of taste	–	Loss of taste	–
Muscle ache	Muscle ache	Muscle ache	Do you have unusual strong muscle pains?
Nausea	Feeling sick (nausea)	–	–
Nausea / vomiting	–	Nausea/vomiting	–
Nose bleed	Nose bleed	–	–
Rash	Rash	–	–
Rhinitis	Runny nose	–	–
Seizures	Seizures	–	–
Shortness of breath	–	Shortness of breath	Are you experiencing unusual shortness of breath? (no; yes mild symptoms/ slight shortness of breath during ordinary activity; yes significant symptoms -breathing is comfortable only at rest; yes, severe symptoms/ breathing is difficult even at rest)**
Sneezing	Sneezing	–	–
Sore throat	Sore throat	Sore throat	Do you have a sore throat?
Vomiting	Vomiting	–	–

It is expected that symptom data from the same PCR-positive cases is captured across the NHS Test and Trace, CIS and CSS datasets. Explicit deduplication of individuals across datasets was not performed but is expected to have no impact on the findings.

The proportion of symptomatic cases varies significantly between datasets, reflecting their different sampling. NHS Test and Trace Pillar 2 and CSS both have the highest number of symptomatic cases, which is not surprising given that both datasets mainly focus on symptom-initiated testing. The NHS Test and Trace SGSS dataset has the next highest proportion of symptomatic cases. We expect to see some asymptomatic screening in SGSS populations, which may explain the decrease in symptomatic cases when comparing Pillar 2 to SGSS. In the CIS study, we see a much smaller proportion of symptomatic cases, likely due to the sampling strategy being independent of symptoms, therefore resulting in asymptomatic and pre-symptomatic individuals testing positive and being included in the study.

**Figure 1:**
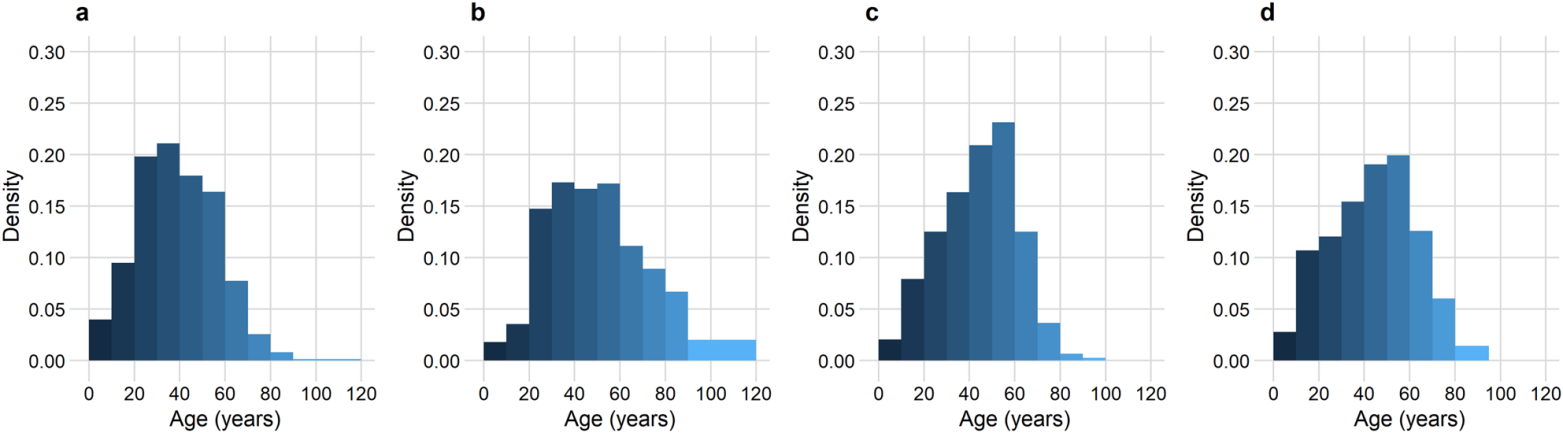
Histograms showing the age density for each dataset. (**a**) Pillar 2, (**b**) SGSS, (**c**) COVID Symptom Study, (**d**) COVID-19 Infection Survey.

The Pillar 2 routine testing contributed by far the largest number of cases to the study, with CIS the fewest. While all datasets contained a slight female majority, with CSS the largest (61.8%), there was some variability in the age distribution of cases (Fig. 1); Pillar 2 routine testing was the youngest, while SGSS included the oldest groups. This is likely to be because SGSS more heavily represents a hospitalised population. CSS and CIS are UK-wide, while NHS Test and Trace data contains cases testing in England.

The characteristics of the infected subpopulation relative to the general UK population have likely changed over time for a multitude of reasons; different levels of restrictions and lockdown across different localities, vaccination coverage and uptake, varying prevalence, weather, levels of outdoors mixing, incentives to ignore social distancing, workplace/school closures and changing availability of testing. Moreover, each study and route of data collection results in different samples of the infected population. The CIS is a population-based household sample and thus should be broadly representative (participation biases aside), those discovered through routine testing (NHS Test & Trace) may overrepresent a population adherent to testing guidance, those prone to more severe infections and the sub-populations with the highest prevalence and testing-seeking behaviour. For the CSS’s app-based reporting, then the sub-populations with high levels of smartphone ownership and compliance are likely to be over-represented.

#### Symptom data

Data is collected at the level of the symptoms experienced by an individual, and for the majority of datasets we have a binary outcome of whether an individual experienced a symptom or not. Exact symptom questions and lists are given in Table 2. In CSS, an individual is able to choose from several levels of fatigue: “none”, “mild” or “severe”. Our planned analyses are designed to work with binary data, and as a result, we map multiple levels into a binary outcome variable. When performing this mapping, we choose to merge levels together, with the aim of making the symptoms as comparable as possible to what is reported in other datasets. Datasets with a binary fatigue variable report 40–60% of cases, which is consistent with most cases only reporting severe fatigue; if we included mild fatigue then we find that close to 80% of cases report fatigue which is inconsistent with what is reported in the other datasets.

#### Symptom reporting windows

The symptom reporting window and its timing relative to the positive test varies across the datasets. For CIS, participants are asked about symptoms in the previous 7 days prior to testing. For cases contacted and interviewed by NHS Test and Trace (Pillar 2 and SGSS), individuals are asked to report symptoms that they are currently experiencing. For CSS, individuals are prompted to report symptoms daily but for this dataset, we include all symptoms reported in the 14 days before and 14 days after the date a positive test is reported (note this does not mean that all participants report symptoms with that level of frequency).

From the time of infection, individuals usually have a few days before they become symptomatic, while test sensitivity also varies over the course of infection, peaking around the time or just before symptom onset. Previous studies have also found patterns in the types of symptoms that present earlier versus later in the course of an infection^11^. Across each of the included datasets, the time in an individual’s infection at which they are tested on average and over which they are asked to report symptoms varies. For CIS time of testing over the course of infection should be random over the period at which someone will test PCR-positive; for data primarily from symptomatic testing, it should be a few days post-symptom onset (reflecting a delay between onset and testing, test result and follow-up interview with Test and Trace). For CSS, the time of testing for many will reflect symptomatic testing in the community and some proportion of individuals with particular symptom reporting patterns are asked to obtain a test through NHS Test and Trace symptomatic testing routes.

#### Symptom classification

To aid interpretation we classify symptoms according to their clinical characteristics. These classifications were made a priori in consultation with an infectious diseases clinician (TW) with experience in caring for people with COVID-19 and without input from observed clustering patterns. We included systemic symptoms, lower respiratory, upper respiratory, gastrointestinal, altered state symptoms and ‘other’ symptoms that did not fit into any of these categories.

#### Research ethics

The secondary analyses described in this paper received ethical approval from the London School of Hygiene and Tropical Medicine (22752). The COVID Symptom Study was approved by the Partners Human Research Committee (Protocol 2020P000909) and King’s College London ethics committee (REMAS ID 18,210, LRS-19/20-18,210) and the CIS received ethical approval from the South Central Berkshire B Research Ethics Committee (20/SC/0195). All methods were performed in accordance with the relevant guidelines and regulations. Informed consent was obtained from all subjects and/or their legal guardian(s).

### Analysis

We describe the frequency with which each symptom was reported in each dataset, categorising them using our symptom classification. We then perform three unsupervised learning techniques, each with a different but complementary aim. Our goal is to understand patterns of symptom co-occurrence and if there is any evidence of symptom clustering, as multiple distinct clusters would be evidence for the existence of distinct COVID-19 symptom phenotypes.

#### Jaccard distance

We use a variety of methods to understand the behaviour of symptoms and the analyses are sometimes performed on the Jaccard distance matrix of symptoms. Letting $\textbf{x}_i$ be the feature vector constructed from the presence or absence of symptom *i* in cases, which has *k*-th element $x_{ik}$, the Jaccard distance between two such vectors is defined as

1 $$\begin{aligned} D_{\textrm{Jac}}(\textbf{x}_i,\textbf{x}_j) = 1 - \frac{\sum _{k=1}^n x_{ik}x_{jk}}{\sum _{k=1}^n (x_{ik} + x_{jk} - x_{ik}x_{jk})} \,. \end{aligned}$$

The simple interpretation of Jaccard distance is then; the proportion of cases who did not experience both symptoms *i* and *j*, given that they experienced at least one of symptoms *i* or *j*. In the case of missing data, the Jaccard distance is computed using only the subset of individuals for which there is no missing data for either symptoms *i* and *j*.

#### Hierarchical clustering

This method starts with a set of symptoms, and the feature vector for each is constructed from their presence or absence in individuals with a positive test and report of at least one symptom (i.e. those positive cases not excluded as asymptomatic). The Jaccard distance is used as an appropriate metric for such binary data. Clusters of symptoms are agglomeratively joined on the dendrogram produced on the basis of the maximum distance between cluster members (called ‘complete linkage’). Symptoms with a low shortest distance between each other on the final dendrogram tend to co-occur, and those with a long distance are not often both present. Clusters can also be identified by ‘cutting’ the dendrogram at a given distance.

#### Logistic PCA (LPCA)

LPCA is an extension of principal component analysis (PCA) to binary data, and reduces the dimension of the symptom space in a manner that preserves the maximum level of variance between individuals (rather than symptoms)^21^. The projection values of symptoms onto lower-dimensional basis are called loadings, and these demonstrate the directions in which individual phenotypes most commonly vary. In practice, the first component is likely to have relatively even contributions from each of the symptoms, and will represent an overall severity of illness at the individual level, with subsequent components demonstrating more subtle ways in which symptoms can vary.

Given an $n\times p$ binary data matrix $\varvec{X} = [X_{ia}]\in \{0, 1\}^{(n\times p)}$, our aim is to find a low dimensional representation of the natural parameter matrix $\varvec{\Theta } = [\theta _{ia}]$, where $\mathbb {P}(X_{ia} = 1) = {{\,\textrm{logit}\,}}^{-1}(\theta _{ia})$. This is achieved by finding $\varvec{{{\hat{\Theta }}}}_k$, a rank-*k* approximation of $\varvec{\Theta }$ such that the Bernoulli deviance, $D_{\textrm{Ber}}(\varvec{X};\varvec{{{\hat{\Theta }}}}_k)$ is minimised. This is conceptually related to logistic regression models, as these also attempt to minimise the Bernoulli deviance. In practice, the minimisation is solved over $\varvec{U} \in \mathbb {R}^{k\times p}$, such that $\varvec{UU}^{\top } = \varvec{I}$, with $\varvec{{{\hat{\Theta }}}}_k = \varvec{UU}^{\top }\varvec{X}$. The column vectors of $\varvec{U}$ are the loadings onto the principal components.

As with all dimensionality reduction techniques, we need to choose the number of dimensions in our low-dimensional approximation. We follow the recommendation of Landgraf and Lee^21^, and examine the change in the Bernoulli deviance as we increase *k*. Consider a rank-0 approximation, where $\varvec{{{\hat{\Theta }}}}_0 = \varvec{1}_n\varvec{{{\hat{\mu }}}}^{\top }$ for ${{\hat{\mu }}} \in \mathbb {R}^n$. That is to say that the natural parameter matrix contains a constant value in every column. This is treated as the null model, to which all other models are compared.

For a model with *k* components, the proportion of Bernoulli deviance explained relative to the null model is given by

2 $$\begin{aligned} P(k) = 1 -\frac{D_{\textrm{Ber}}(\varvec{X};\varvec{{{\hat{\Theta }}}}_k)}{D_{\textrm{Ber}}(\varvec{X};\varvec{1}_n\varvec{{{\hat{\mu }}}}^{\top })} \,. \end{aligned}$$

If $k=p$, then $D_{\textrm{Ber}}(\varvec{X};\varvec{{{\hat{\Theta }}}}_k) = 0$, as the model is saturated and ${{\,\textrm{logit}\,}}^{-1}{\hat{\theta }}_{ia} = X_{ia}$, thus resulting in $P(d) = 1$. This means that *P*(*k*) can be interpreted similarly to standard PCA, in the sense that $P(k)\times 100\%$ of the variance is explained by the first *k* components. The marginal Bernoulli deviance, *M*(*k*), is the change in the Bernoulli deviance explained by adding the $k^{th}$ component, for $k\ge 1$, defined as

3 $$\begin{aligned} M_{\textrm{Ber}}(k):= P(k) - P(k-1) = \frac{D_{\textrm{Ber}}(\varvec{X};\varvec{{{\hat{\Theta }}}}_k) - D_{\textrm{Ber}}(\varvec{X};\varvec{{{\hat{\Theta }}}}_{k-1})}{D_{\textrm{Ber}}(\varvec{X};\varvec{1}_n\varvec{{{\hat{\mu }}}}^{\top })} \,. \end{aligned}$$

When selecting the number of components in our low-dimensional representation of the data, we primarily focus on the marginal Bernoulli deviance and aim to find the largest *k* such that for $k' > k$ the marginal Bernoulli deviance decreases rapidly. We also examine the proportion of Bernoulli deviance explained - if this gets close to 1, then that suggests we have selected too many components and are over-fitting.

In practice, two hyperparameters need to be chosen for logistic PCA: the number of components $k\in \mathbb {N}$, and $m\in \mathbb {R}_+$ which controls the magnitude of the loadings. The optimal choice of *m* varies depending upon *k* and is selected by leave-one-out cross-validation for a range of proposed *m* values.

**Figure 2:**
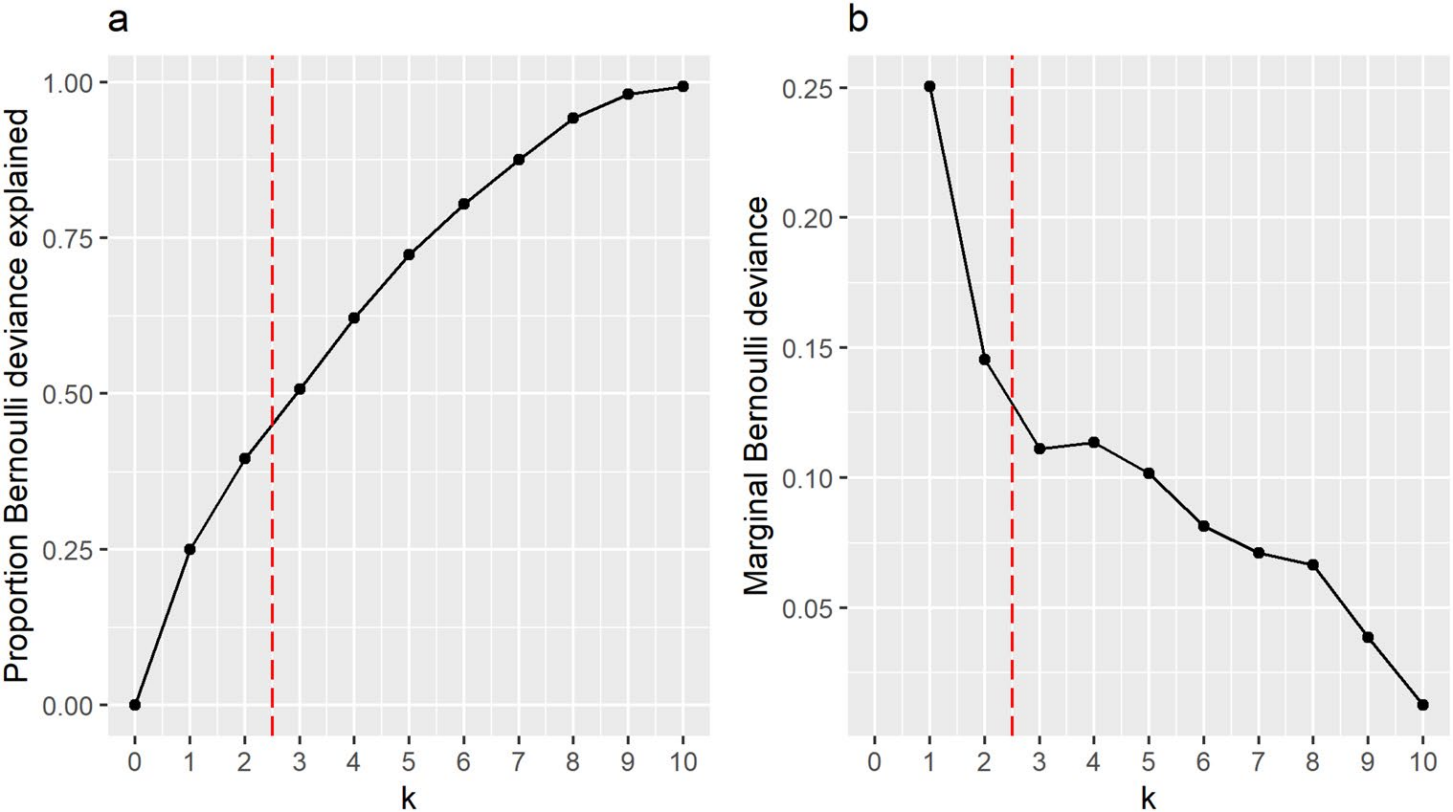
(**a**) the proportion of the Bernoulli deviance explained using an LPCA model with *k* components. (**b**) the proportion of the Bernoulli deviance explained by adding the $k^{th}$ component to the model. In this example, we would select $k=2$ as the true number of components, as indicted by the vertical dashed red line.

An example of the model selection is plotted in Fig. 2. In the plotted example, we would choose ${\hat{k}} = 2$, indicated by the vertical dashed line. This is due to the first two components, having a significantly higher marginal Bernoulli deviance than all models with $k>2$ components. The marginal Bernoulli deviances for models where $k\in \{3,\dots ,8\}$ have small differences between successive values of *k*, making it hard to favour one model over the other. For $k>8$, the marginal Bernoulli deviance does decrease rapidly; however, at this point we have explained close to 100% of the Bernoulli deviance and are overfitting the model at this point. Hence, for this example we choose ${{\hat{k}}} = 2$.

If we select $k=2$ components, then approximately 33% of the Bernoulli deviance of the saturated model is explained. In classic principal component analysis, ideally, the model would find a number of components that explains as close to 100% of the variance while not overfitting to noise. In logistic principal component analysis, however, if a model explains close to 100% of the Bernoulli deviance relative to the null Model, then this is indicative of dramatic over-fitting. For example, the saturated model where $k=d$ will exactly reproduce the input data and explains 100% of the Bernoulli deviance. The true natural parameter matrix will not explain 100% of the Bernoulli deviance, as it tells us about the probability of a symptom occurring; this will lead to a non-zero Bernoulli deviance. As such, our goal is not to explain 100% of the Bernoulli deviance, relative to the null model.

Model selection plots for the number of components ${{\hat{k}}}$ can be found in the code repository for this paper (https://github.com/martyn1fyles/COVIDSymptomsAnalysisPublic). During model selection, we found that in the majority of cases we would definitively choose ${{\hat{k}}} = 2$. In a small number of cases, it was ambiguous whether ${{\hat{k}}}=1$ or ${{\hat{k}}}=2$. In these ambiguous cases, we have opted to take ${{\hat{k}}} = 2$. Our reasons for doing so are: a priori, we believe at least two dimensions are necessary to capture the range of COVID-19 presentations; across all other datasets, we found that ${{\hat{k}}} = 2$, which we believe increases the prior likelihood that ${{\hat{k}}} = 2$ in the ambiguous cases. However, due to this uncertainty, we have made available in the code repository LPCA plots where we have taken ${{\hat{k}}} = 1$. Unlike in traditional PCA, LPCA components are dependent upon the total number of components selected, and as such PC1 for example differs depending upon the total number of components selected. This is why we must rerun the analysis when we take ${{\hat{k}}} = 1$ and present these results separately to results produced where we set ${{\hat{k}}} =2$.

#### UMAP (Uniform manifold approximation and projection)

UMAP is a technique for dimension reduction of complex data based on pairwise distances between symptoms. In contrast to the other methods, it is designed to achieve good separation between unknown classes in the low dimensional space, and as such complements the other machine learning methods used above.

The specifics of the UMAP algorithm are mathematically complex, however, we will provide a brief overview of the algorithms’ strategy. For further details on UMAP, we refer readers to the original UMAP paper^22^ and for practical demonstrations and visualisations we refer readers to^23,24^. The first step of UMAP is to construct an object that describes the shape of the data in high-dimensional space. This object is, effectively, a weighted network where the edge weights represent the probability that two points are connected in the high dimensional space. Points in the high dimensional data are connected, based upon some locally defined notion of distance; if there are regions of the high dimensional space that are dense with points, then the algorithm requires points in that region to be very close when connecting them. If there are regions of the high dimensional space that are sparse, then larger distances are acceptable when connecting points. This locally defined notion of distance is important to ensure that all points in the high-dimensional space form a single connected component. Given this high-dimensional representation of the data, UMAP then attempts to find a network in the low-dimensional space that approximates the representation of the data in the high-dimensional space - this is referred to as an *embedding* and is what we later visualise. Particular attention is given to attempts to preserve the distances between points - but only the distances between points that are connected. By only preserving the most important distances - those between points that are close in the high dimensional space - UMAP is able to produce good low dimensional representations of the high dimensional dataset, particularly when there are complex geometries involved.

The specifics of the UMAP algorithm result in some nuances when interpreting UMAP embeddings. Firstly, the embedding attempts to provide the shape of the data in the high-dimensional space. If the data is clustered in high dimensional space, then the data will also be clustered in the low dimensional space. However, due to the locally varying notion of distance, the compactness of the clusters will not be captured. In addition, if two points in the high-dimensional space are far away from each other, then they will not be connected, and as a result, UMAP will not attempt to preserve the distance between these points. As such, the exact distance between faraway points should not be over-interpreted. The rotation of UMAP plots is not important, only the overall shape of the data. Finally, UMAP is a stochastic algorithm, and there can be minor differences between runs.

To compute our embeddings, we computed the Jaccard distance matrix of symptoms and provided this as an input to UMAP, which we configured to embed the symptoms into a 2-dimensional Euclidean space.

For our datasets, it is also of interest to partition these into different segments of data, such as different age groups, and to compare how the clustering of symptoms changes across each of these segments. Performing this comparison is not straightforward using the base UMAP algorithm, however, as the resulting embeddings can preserve distances of the high dimensional data structure of each segment while looking visually distinct due to rotation or different layouts. To remedy this, AlignedUMAP is an extension of the base UMAP algorithm that attempts to solve this problem by enabling better comparisons between UMAP embeddings of different segments of data. Effectively, AlignedUMAP attempts to find an optimal embedding for each segment of a dataset, and then, as a secondary objective, to minimise the distance between embeddings of adjacent segments in the low dimensional space. This results in embeddings for different segments of data that can directly be compared to each other; we are able to see how distances in the high dimensional space change between segments.

We use AlignedUMAP to produce aligned embeddings for each dataset where we 1) partition the data into three large age bands of key groups (0-17 years, 18-54 years and 55 years), and 2) partition the data into 10-year intervals.

Additionally, we also use AlignedUMAP to align the embeddings produced by different datasets. Each dataset reports a different selection of symptoms, and consequently, the alignment between different datasets is based upon only a core group of symptoms that are shared across all datasets. By aligning the embeddings produced for different datasets, we facilitate easier comparison between datasets, and we can explore if datasets share a common underlying structure.

There are a wide number of UMAP hyperparameters that can be adjusted, and finding the optimal combination is not a solved problem to our knowledge. As part of a sensitivity analysis, we have opted to produce two UMAP outputs for each dataset, one configured to produce a tight clustering of symptoms, and another configured to produce a loose clustering of symptoms. This is achieved by changing the number of neighbouring points that UMAP considers when constructing the high-dimensional representation of the data. As a result, smaller values of the *n_neighbours* parameter will configure UMAP to focus on local structures, and it may not capture the global structure - this produces what we refer to as a tight clustering and produces well-separated clusters. Setting the *n_neighbours* parameter to higher values will configure UMAP to focus less on the local structures of the symptoms but produce a more general clustering of the data – this produces what we refer to as a loose clustering. Both loose and tight UMAP embedding will capture different parts of the symptom topology and produce complementary analyses. When we produce the loose UMAP embeddings that focus more upon the global structure of the data, we take *n_neighbours* = 4, and for the tight UMAP embeddings that focus more upon the local structure of the data, we take *n_neighbours = 2*. When using AlignedUMAP for the fine age strata, we align each segment of data with the two prior segments, and the two slice post the current segments. The numerical sizes of different age segments used are shown in Table 3. We discuss how other UMAP hyperparameters were selected in our Supplementary Materials S1.2, however, we briefly summarise that these other hyperparameters either had less impact on the resulting embeddings, provided that they were not set to extreme values, or could be reasonably selected after due consideration.

**Table 3:** Sample sizes for each strata of the AlignedUMAP embeddings in the main paper.

Age strata	Dataset			
	Pillar 2	SGSS	CSS	CIS
0-9	70,051	2,759	1,256	255
10-19	154,848	3,966	4,891	979
20-29	323,244	16,250	7,716	1,106
30-39	343,935	19,398	10,075	1,416
40-49	292,823	18,673	12,896	1,746
50-59	267,361	19,221	14,263	1,827
60-69	125,840	12,453	7,709	1,153
70-79	41,814	9,963	2,261	552
80-89	12,889	7,488	396	120
90-99	2,222	2,158	-	-

#### Analyses summary

We first run hierarchical clustering, LCPA and AlignedUMAP for all the included cases from each dataset. The UMAP Alignment is performed on common symptoms across datasets for these results, as an attempt to synthesize common structures across datasets.

AlignedUMAP is then run when each dataset is stratified into 10-year intervals and then plotted in 3D space. Here, the alignment is performed across different age strata.

Moving on, we stratify each dataset into the different age groups (0-17 years, 18-54 years and 55 years and older) and rerun hierarchical clustering, LPCA and AlignedUMAP with the alignment performed within the dataset across the three age strata. For only three age strata, it is not necessary to plot the results in 3D space as was required for the results from the finer age strata. Given the large number of plots, the results from our age-stratified findings are presented in the supplementary materials.

### Pre-hoc considerations for comparison across datasets

Because of the different sampling of positive cases and the resulting sample composition, data collection methods, and symptom questions across the datasets, we expect potential differences in findings arising from several causes.

#### Sampling

The majority of routinely detected community cases in the UK were detected via symptom-prompted tests, particularly prior to the widescale availability of rapid antigen testing for asymptomatic individuals in the Spring of 2021. Thus we expect Pillar 2 to over-represent individuals with at least one of cough, fever and loss of taste and/or smell. This bias is also likely to exist within the CSS as a majority of self-reported tests would also have been performed because they met the symptom criteria for routine community testing, though the study also invited a proportion of regular app-user participants to test based on reporting other symptoms. These biases are not present within the ONS study sample.

#### Data collection method

Across all datasets, symptoms are assessed via self-report, including fever. The experience of symptoms and their description is likely to vary across individuals and across demographic characteristics, such as by gender, ethnicity, region, and age. People are likely to report symptoms differently whether they are doing so via an in-person interview, a weekly or bi-weekly survey or via a daily symptom tracking app, and the design of the app or questionnaire interface, as well as the preceding questions, will likely affect reporting. The majority of studies examining the efficacy of symptom self-report have focused on psychiatric disorders. These have generally found agreement between patient self-report and clinician assessment, although this varies from 60% to 90%^25–27^. In major depressive disorder, self-reported symptoms are more severe than clinician-assessed symptoms^25^. When self-tracking for health and fitness purposes, BMI is systematically under-reported^28^. Knowledge of test status could also affect symptom reporting, though this will be less of an issue in the CIS dataset, where individuals will not yet have received their test results. Some studies involve reporting on behalf of others, particularly children or adults receiving care, and communicating the subjective experience of symptoms might be challenging in these cases. When reporting symptoms related to cancer treatment, a dyad (parent and child) approach to reporting symptoms was found to be more effective and preferable to child self-reporting or parent proxy reporting alone^29^.

#### Phase of infection

The symptom reporting window around positive test time varies across the different datasets. There is evidence from previous studies^11^ that some symptoms tend to appear earlier in infection while some appear later. We also know that people who test negative, who are not included in this dataset, report a wide range of symptoms that are not related to SARS-CoV-2 infection^12^; widening the symptom reporting window around a test date might include symptoms that are non-specific to the SARS-CoV-2 infection. Our approach collapses across time and these variations in the reporting window could affect our findings regarding symptom frequency and clustering. While there is no way of varying this for the routinely collected NHS Test and Trace data, we do conduct sensitivity analyses to examine a wider symptom reporting window around the day of testing for the ONS dataset, making it more comparable to CSS. We arbitrarily define positive episodes as a new positive occurring more than 90 days after an index positive or after 4 consecutive negative tests and consider symptoms reported in [-7,+35] days around the index positive. We do not find that this wider symptom window affects our clustering and co-occurrence findings.

#### Epidemic phase

The characteristics of cases differ over the course of the epidemic, for example by age, region, socioeconomic characteristics or variant of SARS-CoV-2 infection, which in turn could plausibly affect the symptoms experienced and the likelihood that they are reported. Some positive cases could be from single or double-vaccinated individuals, particularly from later time periods in Winter/Spring 2021. Similar to our AlignedUMAP embeddings for age-stratified data, we could also produce AlignedUMAP embeddings for time-stratified data, allowing us to investigate how symptom co-occurrence patterns change over time. This would be of particular interest as vaccination effects build, or as a new variant with a different disease profile becomes dominant. The requirement of such an analysis is that each time strata has a sufficient number of points such that the estimated Jaccard distance matrix is not subject to significant uncertainty. An initial exploration of this analysis was performed for Pillar 2 and SGSS datasets, by stratifying into week-long strata; however, no significant changes to the symptom co-occurrence patterns were observed during this time period.

## Results

### Hierarchical clustering

**Figure 3:**
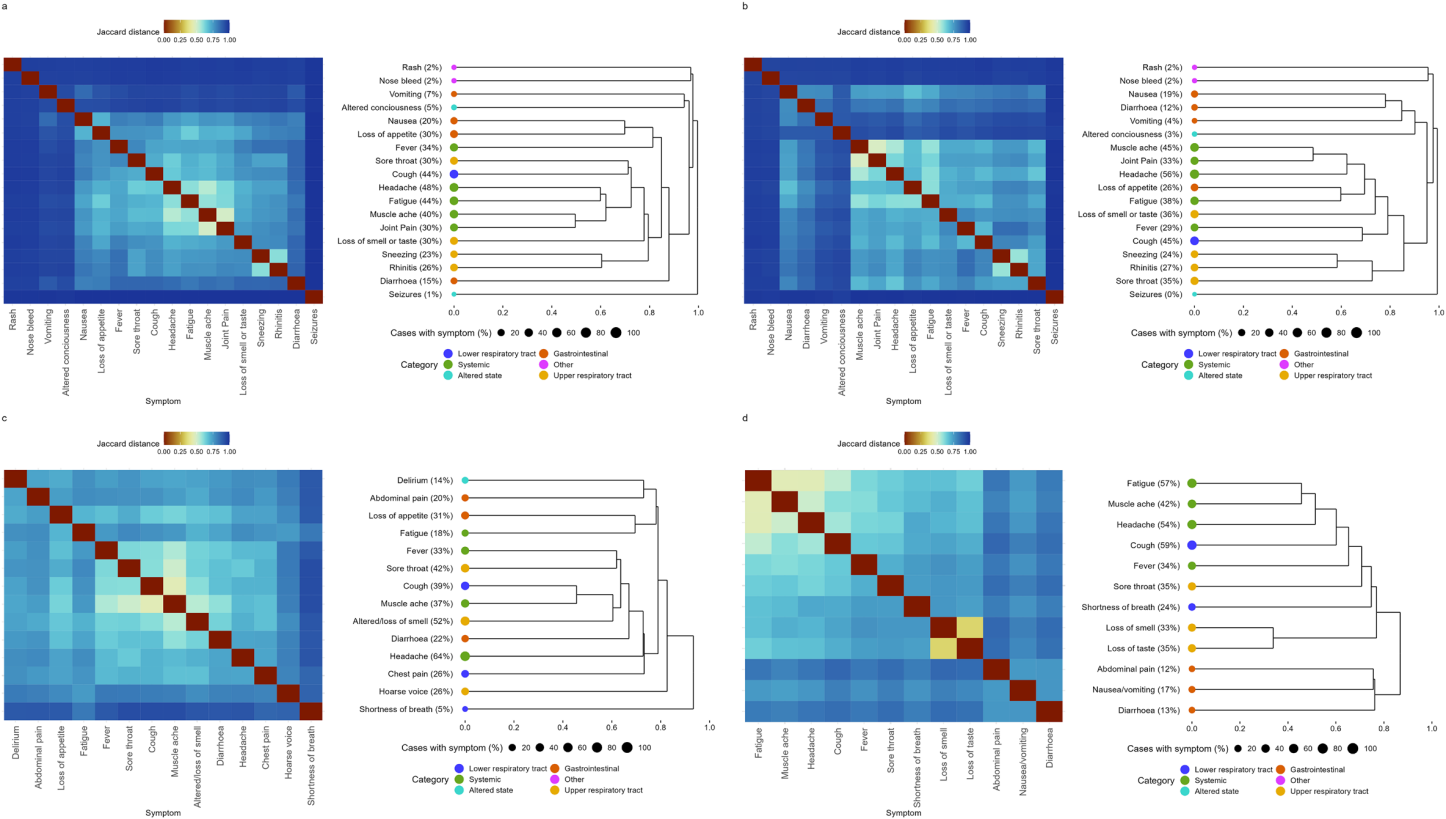
Jaccard distance matrices between symptoms adjacent to associated dendrograms obtained through hierarchical clustering under complete linkage. The symptom category is denoted using coloured points at the roots of the dendrogram. The central columns give the name of the symptom with the percentage of symptomatic cases who exhibit the symptom in the dataset. (**a**) Pillar 2, (**b**) SGSS, (**c**) COVID Symptom Study, (**d**) COVID-19 Infection Survey.

We first performed hierarchical clustering using complete linkage^30^ and the Jaccard distance between symptom vectors as defined in Equation (1), with results shown in Fig. 3. This figure shows the matrix of such distances as a heatmap, with a dendrogram to its right. We read these dendrograms from right to left, with splitting points representing points at which the algorithm suggests a separation of symptoms into groups on the basis of their occurrence in infected individuals.

Of the plots, the CIS data in panel d shows the clearest signal of separation of symptoms under this analysis method: gastrointestinal symptoms form a separate symptom grouping, joining the rest of the hierarchy only at the highest level; the distinctive loss of taste and smell joins the tree at the next; and the remaining symptoms join individually at remaining levels. In Pillar 2 and SGSS data (Fig. 3 panels a and b) a similar pattern is observed, except for additional complexity associated with uncommon symptoms in $\le 5\%$ of positives and for Pillar 2 loss of smell or taste joining at a similar point on the tree to upper respiratory tract symptoms. For the CSS data in panel **c**, we see that shortness of breath and hoarse voice, symptoms not collected in other studies, appear before gastrointestinal symptoms join the tree.

### Logistic principal component analysis

**Figure 4:**
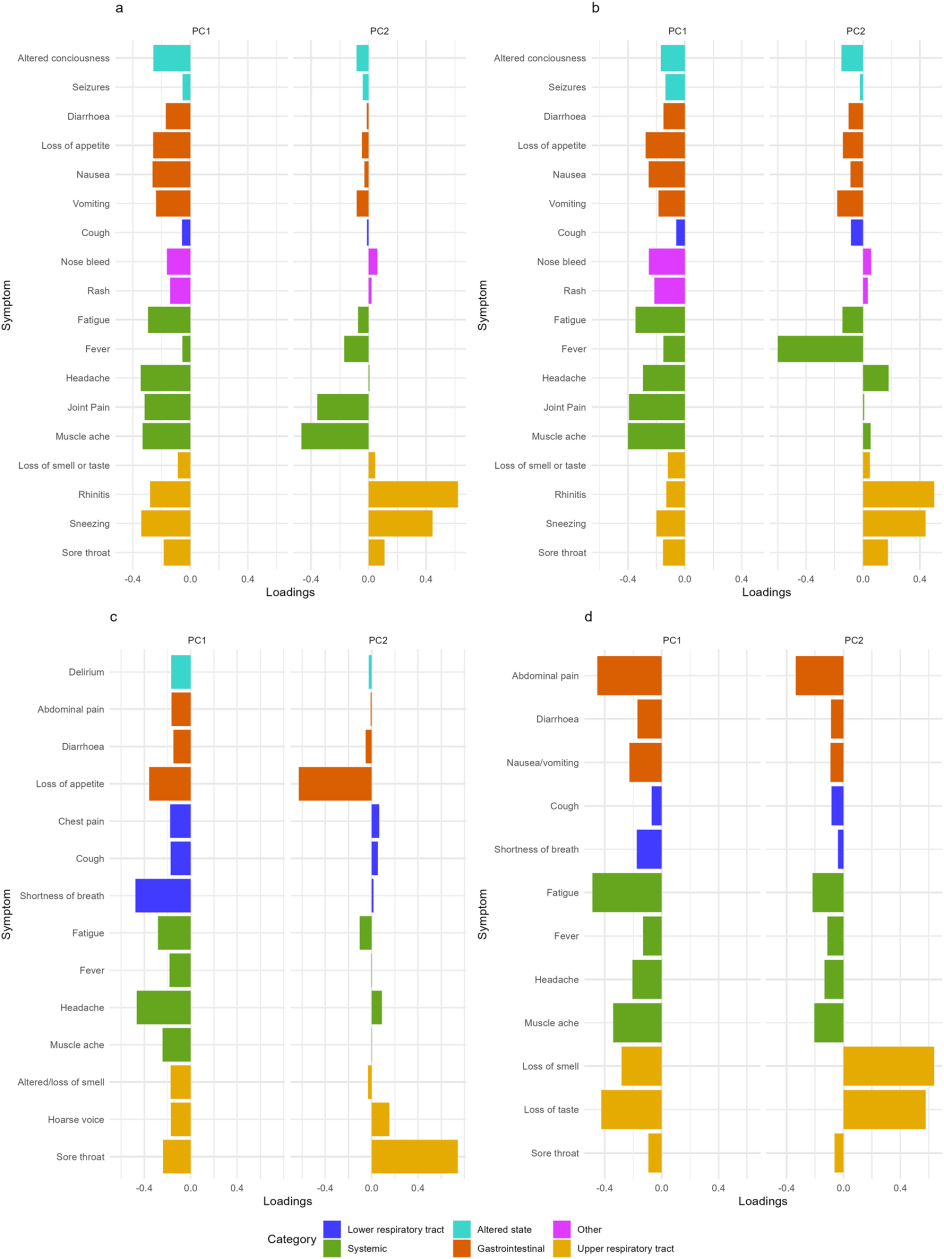
Logistic Principal Components Analysis (LPCA) results. For each dataset, elements of the principal components are visualised as vertical bar plots. Each vector is insensitive to overall multiplication by $-1$. Symptom categories are labelled by colours. (**a**) Pillar 2, (**b**) SGSS, (**c**) COVID Symptom Study, (**d**) COVID-19 Infection Survey.

Secondly, we performed Logistic Principal Component Analysis (LPCA), an extension of Principal Component Analysis to binary data^21^. This method is used to project the set of individual feature vectors $\{\textbf{y}_i \}$ for each dataset onto (in our case two) components that sequentially are as close to the original set of vectors as possible. The results of this analysis are shown in Fig. 4, and show quite strikingly consistent patterns across datasets, despite the various biases and data collection techniques employed.

The first strong signal in the data is that the first principal component involves all symptoms in the same direction, meaning that the closest one-dimensional description of community symptoms is the number of symptoms experienced. The second principal component, with some exceptions that vary by dataset, suggests that a source of variation is a negative correlation between upper respiratory tract symptoms and systemic (Pillar 2, SGSS and CIS) and gastrointestinal symptoms (SGSS, CIS and CSS). The overall interpretation of these results show that a parsimonious description of COVID-19 symptoms at the individual level can be provided by quantifying the total number of symptoms experienced, followed by the relative contribution of upper respiratory symptoms versus systemic or gastrointestinal symptoms to the total number of symptoms experienced. The contribution of upper respiratory versus systemic and gastrointestinal symptoms is also seen and in fact, strengthened when examining the age-stratified data (children 0-17 years, adults 18-54 years and elder adults aged 55 years and older, see Supplementary Materials).

### Uniform manifold approximation and projection

Having different symptoms identified by taking a symptom-level view of clustering as in the hierarchical analysis, and an individual-level view of co-occurrence as in LPCA, is explained by questions these methods address. LPCA attempts to find a description of the overall variation of the symptoms of individuals within the dataset, while hierarchical clustering groups by suitably defined co-occurrence to find natural clusters of symptoms within the dataset. Our third main analysis method aims to provide an overall picture by considering low-dimensional embeddings of the data based on the structure of interactions encoded in the datasets. In particular, Uniform Manifold Approximation and Projection (UMAP) and associated algorithms^22,23^ produces a low-dimensional embedding using the local structure of the data (i.e. groups of commonly co-occurring symptoms) and provided the intrinsic dimension of the system is not too large, and can capture some of the global structure of the data (i.e. the relationships between such groups of data points). The result is that symptoms which commonly co-occur are placed close to each other in the outputted low-dimensional embeddings. Hyperparameters are important for UMAP, so we performed the analysis for two different hyperparameter choices: one that focuses more on the global structure (shown in Fig. S2); and one that focuses less on the global structure and attempts to preserve more of the local structure of the data (shown in Fig. S3).

To more explicitly compare findings across datasets, we extend the UMAP analyses above by using the AlignedUMAP algorithm^23^. AlignedUMAP takes several different datasets as inputs and finds the optimal embedding for each inputted dataset, subject to the loose constraint that data points that are shared between datasets are placed in similar positions in the low-dimensional embeddings. These are produced through a trade-off between finding the optimal embedding for individual datasets, and aligning the embedding of shared symptoms across datasets. By aligning embeddings, we gain several useful insights, most importantly that an embedding can be directly compared with the others it was aligned against, allowing better assessment of similarities and differences.

**Figure 5:**
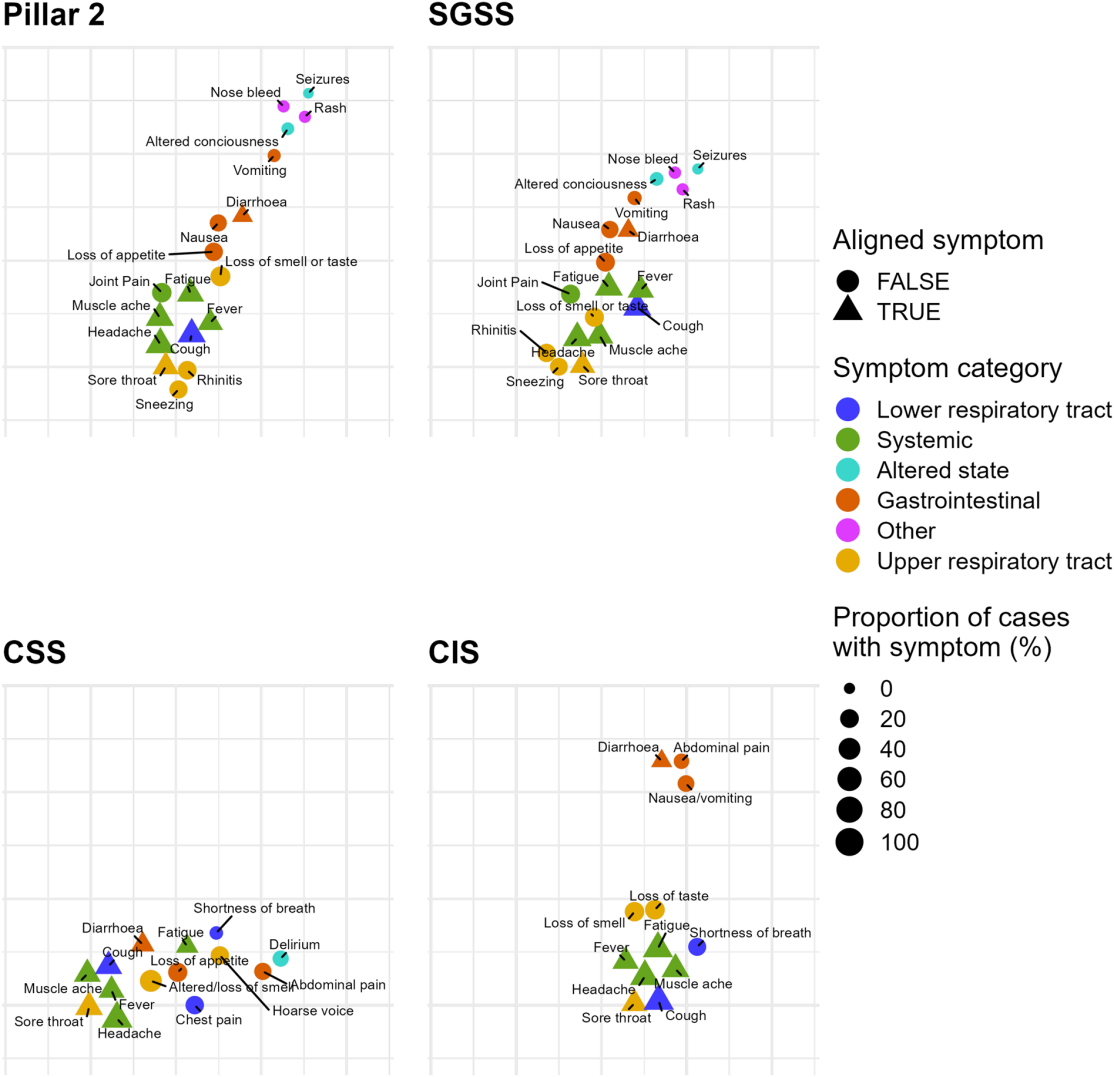
AlignedUMAP embeddings of SARS-CoV-2 symptoms. For each dataset, an optimal embedding of the symptoms into 2D Euclidean space is found, subject to the following loose constraint: if a symptom is common to all datasets, then it should be placed in roughly the same position across all datasets. This alignment allows for easier comparison, and investigation of shared symptom structures across all datasets. Point size is proportional to the proportion of cases that develop a given symptom. Symptoms that are common to all datasets, and are aligned between distinct datasets are plotted as triangles. For this embedding the parameters were chosen to capture more of the global structure of symptoms and produces less well-defined clusters. (**a**) Pillar 2, (**b**) SGSS, (**c**) COVID Symptom Study, (**d**) COVID-19 Infection Survey.

We produce embeddings of each dataset that are aligned based on the core symptoms shared by all the datasets in our analysis: cough, diarrhoea, fatigue, fever, headache, muscle ache, and sore throat. These, shown in Fig. 5, allow us to explore whether datasets shared a common underlying structure of symptom co-occurrence.

Inspection of the embeddings with alignment based on the core symptoms shared by different datasets provides some evidence of a broad structure shared across all datasets. The embeddings produced can be broadly described by a central cluster of systemic symptoms, and cough. Lower respiratory tract symptoms are typically placed nearby, in particular with shortness of breath often being placed close to fatigue. The upper respiratory tract symptoms (sore throat, rhinitis, sneezing) are typically placed further away from gastrointestinal symptoms, with the exception of lost/altered smell or taste symptoms. On most plots, the gastrointestinal symptoms exist as a tail or are slightly separated from the main central group of systemic symptoms. The main exception to this is the CSS data, although we would caution against over-interpretation of this plot since, in contrast to the other studies, CSS has a more diverse set of symptoms, meaning that the task we have set of aligning the symptoms common to CSS and other datasets, at the same time as preserving relationships between those symptoms and ones unique to CSS, is inherently challenging. Overall, therefore, we argue that the UMAP results complement the LPCA analysis, which suggested that individuals separate between those who experience upper respiratory tract symptoms or those who experience a mixture of systemic and gastrointestinal symptoms.

**Figure 6:**
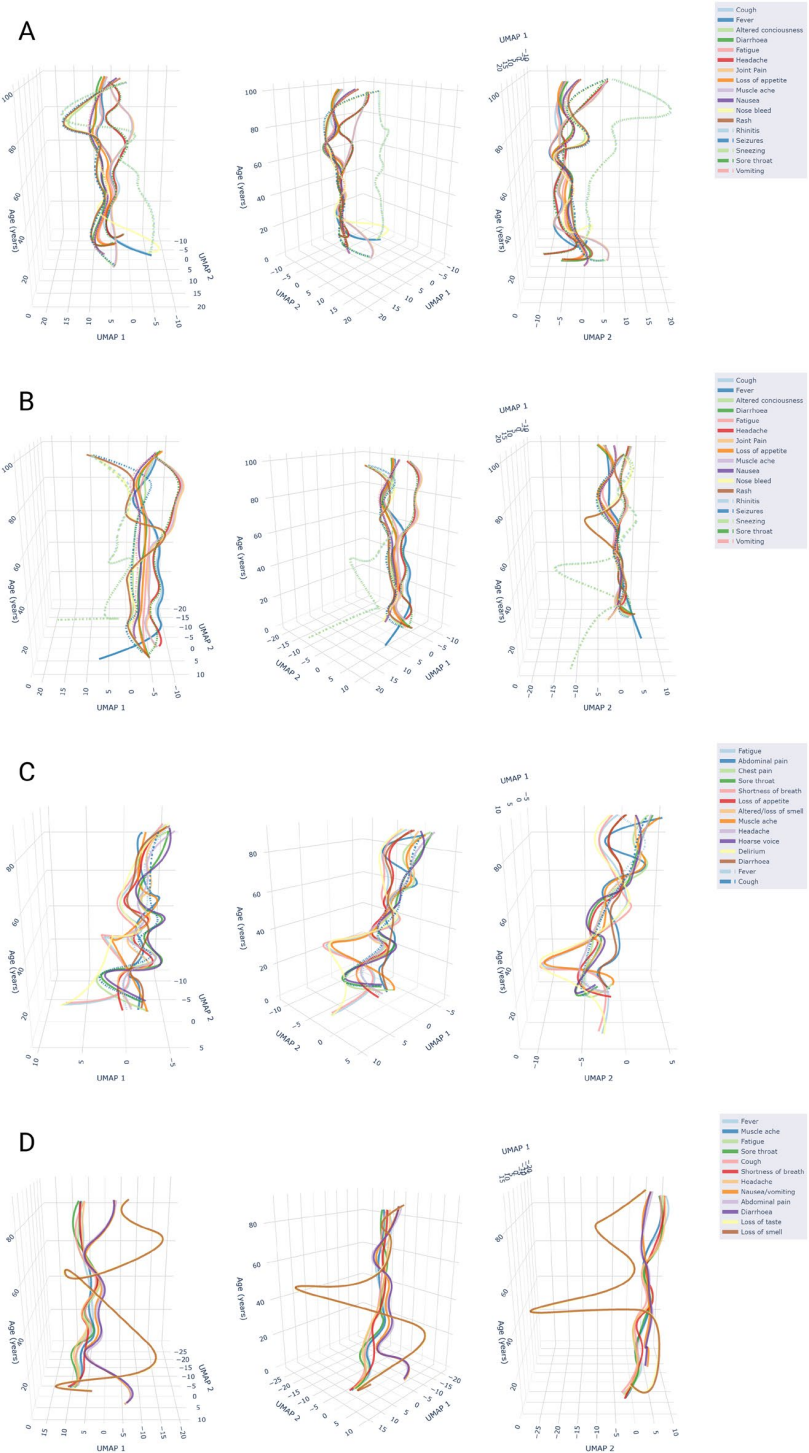
AlignedUMAP embeddings of SARS-CoV-2 symptoms across several datasets. Each dataset has been age-stratified into strata of length 10 years. For each strata, an optimal two-dimensional embedding into Euclidean space of the symptoms is found, subject to the loose constraint that each symptom is placed in a similar location in adjacent embeddings. Linear interpolation is used to connect the embedding of each strata, allowing for a 3-dimensional visualisation of how the co-occurrence patterns of symptoms change with age. For each 3D embedding, we take three images at 45 degree rotations. (**a**) Pillar 2, (**b**) SGSS, (**c**) COVID Symptom Study, (**d**) COVID-19 Infection Survey.

As we did with hierarchical clustering and LPCA, we stratified each dataset based on age bands that represent children and adolescents, adults and elders, and produced aligned embeddings for ease of comparison (see Supplementary Materials). However, AlignedUMAP allows us to directly compare more embeddings than is possible for dendrograms or symptom loadings, as there exist explicit relationships between the embeddings. We perform an additional analysis where we again age-stratify each dataset into 10-year strata and produce aligned embeddings. These embeddings can then be visualised in 3-dimensional space to describe how patterns of symptom co-occurrence change as age increases, see Fig. 6, where linear interpolation has been used to connect the different embeddings from each ten-year age strata. In supplementary Figures ??, we also provide 2D marginal plots of the embeddings. Across all datasets, we observe changes to the local structure, indicated by the splitting of the rope/ribbon-like structures for the youngest age strata (under 10 years old), and for the older age strata (around 70 years old). The changes indicate that, despite the attempt to align symptoms in adjacent embeddings, the symptom-co-occurrence patterns of the data have changed too substantially for that to be achieved.

This is clearest in the CIS dataset, where some gastrointestinal symptoms (diarrhoea, nausea/vomiting, abdominal pain) are separated out from the main body of symptoms for the youngest and older age strata. In Pillar 2 and SGSS, we find the formation of new clusters of symptoms, in the older age strata with a first cluster containing vomiting and nausea, and a second cluster containing headache, sore throat, muscle ache and joint pain. For the CSS dataset, separation into two main symptom clusters is observed, with one cluster containing: abdominal pain, muscle ache, headache, sore throat, chest pain, and cough, and with the second cluster containing loss of appetite, altered/loss of smell, diarrhoea, hoarse voice, slightly separated shortness of breath, fever, delirium and fatigue.

For the under-10s, the produced embeddings typically consist of small clusters of symptoms. The CIS dataset is the exception by again separating out gastrointestinal symptoms from the main body of symptoms. Inspection of the Jaccard distance matrices for the youngest age strata suggests that a possible explanation may be that fewer total symptoms are reported for young children. The observed clusters in the embeddings appear to consist mainly of pairs, or triplets of symptoms that do commonly co-occur, e.g. rhinitis and sneezing. However, the level of co-occurrence between these distinct small clusters is very small, leading to separation in the low dimensional embeddings.

## Discussion

In summary, we have shown that considerable complexity and variation exists in COVID-19 symptoms in community infections. We find that the primary source of variation is in the number of symptoms experienced by a case, but conditional on this there are various ways to be ill that provide a more fine-grained description of phenotypes. In particular, we find evidence for the separation between upper respiratory and systemic symptoms, both including commonly reported symptoms, and between upper respiratory and gastrointestinal symptoms, though the latter is less common overall. While the deep structure of the symptom clustering was similar across the middle range of age groups, we found some evidence that patterns of symptom reporting changed among the youngest and oldest, though further work may be required to understand whether this is due to symptom reporting differences, or differences in the symptoms experienced.

While there are some differences in our findings across the four datasets, this is unsurprising given their different case sampling designs, data collection methods, symptom reporting windows and specific symptom data collected. Routinely tested cases, for instance, will be selected based on the symptoms that qualify cases for testing (Pillar 2), leading to lower expected variation in the presence of these symptoms compared to cases identified via random sampling. Indeed, the broad consistency of findings across these datasets, which derive from routine, representative household and participatory surveillance methods respectively increases our confidence that our findings are robust.

Our findings have implications for case identification and associated public health guidance in the community, particularly school settings, and high-risk settings such as care homes or hospitals. The existence of phenotypes would suggest that the one-size-fits-all symptom-based criteria for symptomatic testing like that used in the UK from 2020-2022 may be sub-optimal, especially in these sub-populations where multiple phenotypes are most likely. Differences by age could imply that symptomatic testing criteria should be tailored for different settings, though this would need to be balanced with what is feasible and understandable for the public. Further, it may be the case that the different characterisation of cases could inform clinical outcomes, for example the finding that cases can be described by the contribution of upper respiratory symptoms versus systemic or gastrointestinal symptoms to the total number of symptoms experienced. We find that the symptom clustering patterns amongst the oldest age groups diverged from the middle age groups, which is of potential clinical relevance given the strong age-related risk of severe disease.

Routinely collected datasets in this study include symptom information only from positive SARS-CoV-2 cases, meaning that we cannot evaluate the specificity of symptom testing criteria combinations informed by the symptom co-occurrence structures we have identified here, and this limits our direct evaluation of the symptomatic testing policies that were employed during 2020-2022. However, studies that examined the optimal combination of symptoms to initiate testing of symptomatic community cases^12,13^ may have been implicitly assuming the existence of a single phenotype - to ensure that a symptom testing criteria is optimal, the possible existence of multiple phenotypes and the wide spectrum of disease must be considered. Emphasis should be placed on the extent of symptom variation across COVID cases in communication with the public. This messaging is critical for the initiation of transmission control interventions, including isolation, and in helping the public to manage risks, including transmission to more vulnerable contacts. Given that our datasets only consist of positive SARS-CoV-2 cases, we are unable to explore the clustering of symptoms in the “background” landscape of symptoms that are caused by other infections, allergies or environmental conditions. Such an analysis could further elicit whether there are clusters of symptoms that are well-distinguished from other background symptoms and could further inform optimal symptom testing criteria, however, we leave this as future work given that the majority of datasets analysed here do not contain data on individuals testing negative for SARS-CoV-2.

As well as optimising response with respect to symptoms upon acute infection, another key question involves the role of comorbidities, including chronic conditions^8^. Unfortunately, information on these is not collected in as consistent and systematic a manner across large datasets as information on short-term symptoms. As such, adjustment for comorbidities is not possible without major additional data collection and/or linkage, which would be likely to be a fruitful direction for future research, for example, using the secure linkage methodology of Williamson et al.^7^.

With vaccination, re-infections and ongoing SARS-CoV-2 evolution, as well as the resurgence of other previously suppressed respiratory infections, understanding the variability of COVID-19 symptoms presentation is critical in planning community intervention for control of transmission, identification of cases potentially requiring greater care, and possibly understanding long term presentation of the disease^31^. Beyond even the current pandemic, the application of unsupervised learning analyses, such as this one, in conjunction with clinical, epidemiological and behavioural understanding is likely to yield important insights for other infectious diseases.

### Supplementary Information

Supplementary Information.

## Data Availability

Datasets are too sensitive for public release, and can be accessed by researchers through secure research environments. The first of these is the Secure Anonymised Information Linkage (SAIL) Databank, with information for researchers wishing to access this resource at https://saildatabank.com. The second of these is the Office for National Statistics’ Secure Research Service (SRS), with information for researchers wishing to access this resource at https://www.ons.gov.uk/aboutus/whatwedo/statistics/requestingstatistics/secureresearchservice. Code and datasets that have been approved for publication are available at: https://github.com/martyn1fyles/COVIDSymptomsAnalysisPublic.
