# Supplementary material for: Diversity of symptom phenotypes in SARS-CoV-2 community infections observed in multiple large datasets
Source: Sci Rep. 2023 Dec 7;13:21705. doi: 10.1038/s41598-023-47488-9 (PMC10709437; doi:10.1038/s41598-023-47488-9)
Supplement: Supplementary file 1 — Supplementary Information. [file 41598_2023_47488_MOESM1_ESM.pdf]

Supplementary Materials for:

Diversity of symptom phenotypes in SARS-CoV-2 community infections observed in multiple large datasets

Martyn Fyles Karina-Doris Vihta Carole H Sudre Harry Long
Rajenki Das Caroline Jay Tom Wingfield Fergus Cumming
William Green Pantelis Hadjipantelis Joni Kirk Claire J Steves
Sebastien Ourselin Graham Medley Elizabeth Fearon Thomas House

S1 Supplementary Text

S1.1 Symptom frequencies

All datasets include only cases reporting at least one symptom for these analyses. The most commonly reported symptom across all datasets was headache, with approximately half of the cases in the Pillar 2, SGSS and CIS datasets reporting them, and almost two-thirds of those from CSS, (see Figure 3 from the main text and Figure S1 below). The frequency of systematic symptom reports is high across the datasets. Fever, a systemic symptom intended to prompt isolation and testing in the UK, was experienced by less than one-third of all symptomatic cases. Cough, another isolation and testing initiating symptom (when new and continuous, which was not captured in these datasets), was also common (39% to 59%). NHS Test and Trace did not include any other lower respiratory tract symptoms, but in CIS, shortness of breath was experienced by 24% and by 5% in CSS, while 26% of those symptomatic cases participating in CSS reported chest pain (not collected in other datasets). Each dataset includes information about altered/loss of smell and/or taste but collected this differently, though all variations were commonly reported. Altered/loss of smell was most frequently reported in the CSS (52%), while loss of taste and smell separately (CIS) and in combination (NHS Test & Trace) was reported by over 30%. These symptoms also trigger isolation and testing. Sore throat was a common upper respiratory symptom in all datasets (30% to 42%). Sneezing and rhinitis, only collected by Test and Trace, were reported by around one-quarter of symptomatic cases. Gastrointestinal symptoms tended to be less frequent than systemic and respiratory but were not unusual (mainly reported by 10-20% though less frequently for vomiting alone), with the exception of loss of appetite, which was reported by between one-quarter and one-third of cases in Test and Trace and CSS, datasets in which it was collected. Symptoms that we described as 'altered state' were rarer and not collected in CIS. Rash and nosebleeds were reported by 2% of symptomatic cases in Test and Trace, but not collected in CIS or CSS.

S1.2 UMAP Hyperparameter Selection

The base UMAP algorithm has four main hyperparameters; `n_neighbours`, `n_components` and `min_dist`. In the main text, we discuss how we have chosen to vary `n_neighbours` between

two values to produce "loose" (`n_neighbours` = 4) and "tight" (`n_neighbours` = 2) clusterings, which focus more on either local or global structure. These two values of `n_neighbours` were selected as they produced distinct embeddings that demonstrated different aspects of the high-dimensional data structure. More extreme values of `n_neighbours` produced embeddings that did not appear to perform well at describing the structure of the data.

The number of dimensions of the embedding is set by n_components, which we have fixed at two dimensions. During the development of this paper, we explored using 3D embeddings however we found that they were not sufficiently different to the 2D embeddings to warrant their inclusion, particularly given the increased challenge of visualising a 3D embedding in a paper. The next parameter is min_dist, which provides a minimum distance between points in the produced embeddings. The role of this parameter is largely to improve the readability of UMAP embeddings by preventing points from being overplotted in embeddings which can make them difficult to read. We find that setting this parameter to large values can impact the quality of the embedding by artificially enforcing large distances between points. Therefore to set this parameter, we started off with the smallest possible value and increased until the produced embeddings were sufficiently readable. Finally, we configured our UMAP algorithm to embed into a Euclidean space, and while it is possible to vary this, we did not believe that embedding to a non-Euclidean space would help elicit further information.

AlignedUMAP inherits all the parameters of the base UMAP algorithm, and we apply the same arguments for how to set these parameters. In addition, AlignedUMAP has two new hyperparameters; `n_slices` and `alignment_strength`. Recall that AlignedUMAP first produces embeddings for different segments of a dataset, such as different age groups, and then attempts to minimise the distance between the embeddings between different segments of the data to produce embeddings that can directly be compared for different segments of the dataset. Assume without loss of generality that a dataset can be partitioned into ordered segments $S_1, S_2, S_3, \ldots$. The parameter `n_slices` controls how many of the neighbouring segments with be used in the Alignment process. For example, if `n_slices` = 1, then S_i will be aligned with S_{i-1}, S_{i+1}, it's immediate neighbours. If n_slices = 2, then S_i will be aligned with $S_{i-2}, S_{i-1}, S_{i+1}, S_{i+2}$, it's two neighbours on either side. The effect of increasing `n_slices` is to increase the smoothness of the AlignedUMAP embedding, akin to increasing the width of a histogram bin. For large values of `n_slices`, the AlignedUMAP embeddings are over-smoothed, and for small values of `n_slices` the embedding is under-smoothed. Additionally, it would not make sense to align the 0-10 age group with the 50-60 age group, where we would expect there to be different symptom occurrence patterns. Therefore, we prefer a smaller value of `n_slices` to prevent this. We find that `n_slices` = 2 is an ideal value that demonstrates the varying changing symptom occurrence patterns. At `n_components` = 1, the embeddings are very noisy and at `n_components` ≥ 3, the embeddings are over-smoothed into a single cluster of symptoms across all embeddings. Performing AlignedUMAP necessitates a small trade-off between finding the optimal embedding for a given segment, and aligning it with its neighbours - this trade-off is controlled by the `alignment_strength` parameter. For this parameter, we generally prefer small values to ensure that we are still finding the optimal embedding for each segment of data, with the additional benefit of the embedding being aligned with the embeddings of adjacent segments. Therefore, to set this parameter, we slowly increased the value of this parameter until the embeddings were aligned, via visual inspection.

We believe that these are suitable methods for setting the UMAP parameters. For readers that are interested in exploring the effects of these parameters further, we provide the necessary data and code in our repository to reproduce this analysis.

S1.3 UMAP results without alignment between datasets

Looking at Figure S2, we see a global structure similar to what we observe in the main paper using the AlignedUMAP algorithm. The embeddings of most datasets can be described by a central cluster of systemic and lower respiratory tract symptoms. Upper respiratory tract symptoms, such as rhinitis, sneezing, hoarse voice, and sore throat, are typically placed close to the systemic symptoms cluster, with the exception of loss of smell and taste symptoms. Gastrointestinal symptoms are often placed further away from the upper respiratory tract symptoms and often form a tail leading to some of the rarer symptoms. We note that these embeddings synthesise the results we observed from the LPCA loadings, where the second loading suggested that cases could be separated based on whether they predominantly experienced upper respiratory tract symptoms or systemic and gastrointestinal symptoms. The relatively low rates of occurrence of gastrointestinal symptoms explains their appearance high in the hierarchical tree, while the higher frequency of systemic and respiratory symptoms explains their relative importance in LPCA loadings, within the general structure revealed by UMAP.

We repeat the UMAP analysis without alignment, this time with the algorithm tuned to focus more on the local structure of the data and less on the global structure of the data. As shown in Figures S3, this produces a better separation of the symptoms into clusters in the low dimensional embeddings, however, some of the relationships between these clusters may be lost. In the resulting embeddings, we observed several pairs of symptoms that commonly co-occur but appear to be distinct from the main cluster of other symptoms, notably sneezing and rhinitis in the Pillar 2 and SGSS datasets, headache and sore throat in the CSS dataset, and loss of smell and taste in the CIS dataset. The remaining symptoms are often packed into two tight clusters. For Pillar 2 and SGSS, a clear separation between systemic and upper respiratory tract symptoms, and the less frequently occurring gastrointestinal, altered state and other symptoms is observed. Similarly, gastrointestinal are placed into their own cluster in CIS, and in CSS with the exception of loss of appetite. Focusing more on the local structure can make the resulting embeddings more variable between datasets, as the choice of symptoms included in the dataset appears to make more of a difference. We note that the embeddings focusing more on the local structure can be more variable between repeats, however, they do highlight small local structures in the data. The aligned UMAP results in the main paper focus more on local structure, however, the requirement to align several related slices of the datasets appears to make these results more consistent between runs.

Looking at Figures S2 and S3, we see a global structure to the relationship between symptoms that synthesises other results. This is clearest in the CIS data, where we can draw a line from gastrointestinal through systemic, to respiratory tract symptoms, but with sore throat closer to cough than it is to loss of taste and smell. Such a line could be interpreted as describing a spectrum of COVID-19 symptoms. In the other datasets, this pattern is complicated by other types of symptoms, which typically occur closest to gastrointestinal. The relatively low frequency of these symptoms explains their appearance high in the hierarchical tree, while the higher frequency of systemic and respiratory infections explains their relative importance in LPCA components within the general structure revealed by UMAP.

S1.4 Age stratified findings

We repeated our main analyses - hierarchical clustering, Logistic PCA and AlignedUMAP - on each dataset, stratified by broad age groups: children (0-17 years), adult (18-54 years) and elder adults (55+ years), Supplementary Figures S4-S19.

Broadly, we did not find strong differences in the clustering and co-occurrence patterns of symp-

toms across age groups and studies. The unstratified findings reflect more strongly the middle age category (18-54 years), which accounts for the majority of the sample in each dataset. It is possible that symptom data collection, particularly among young children, which relies upon caregiver reports, could contribute to explaining some differences observed.

The clear separation of gastrointestinal symptoms and loss of taste and smell is observed across the age strata in the CIS, Supplementary Figure S7, with minor differences in the order at which some other individual symptoms join the tree (e.g. shortness of breath among children and sore throat amongst elder adults). In Pillar 2 and SGSS datasets, Supplementary Figures S4 and S5 respectively, across age groups the rarer symptoms separate earlier from other symptoms, with some later separation between systemic and upper respiratory symptoms observable. Patterns did not differ greatly across the age strata. Across age strata, symptoms among cases in the CSS, Supplementary Figure S6, show shortness of breath and delirium (rare symptoms) separating early, followed by some gastrointestinal symptoms (diarrhoea and abdominal pain) and, most clearly among adults 18-54, splitting between systemic and gastrointestinal symptoms and primarily lower and upper respiratory symptoms.

For all age-stratified LPCA analyses plotted in Supplementary Figures S8-S10, the first principle component essentially describes variation in severity, followed by characterisation according to either upper respiratory (loss of taste and smell) or upper respiratory symptoms. For CIS, plotted in Supplementary Figures S11, cough had a high loading on the second component among children but not adults or elder adults, pointing the opposite direction to upper respiratory symptoms. The presence of gastrointestinal symptoms was more important in describing cases among elder adults, compared to children, with adults aged 18-54 years in between.

Similar patterns of separation between upper respiratory, systemic and gastrointestinal symptoms are seen across age groups when examining the UMAP embeddings when hyperparameters were selected that produce well-separated clusters, Supplementary Figures S16-S19. Despite the age strata being coarser here than in the results of the main paper, Fig 4, we do observe similar structural changes to the data: in the children's age strata, we often observe the formation of several small clusters of symptoms; in the adults' age strata, the embeddings tend to resemble a larger cluster; and in the elders' age strata, the embeddings again start to fragment into two smaller clusters of symptoms. The structural changes are less striking than in the results in Fig. 4, where finer age slices are used. However, this is expected, given that the coarser age strata used in Supplementary Figures S16-S19 make it harder for UMAP to detect structural changes to patterns of symptom co-occurrence that occur over small changes in age.

The results from tuning the UMAP algorithm to focus more on global structure are plotted in Supplementary Figures S12-S15. Unlike in embeddings that focus more on the local structure of the dataset, we do not observe a strong separation of symptoms into several small clusters in the youngest or separation into two main clusters in the elderly population. This is to be expected, as focusing more on the global structure results in an embedding that attempts to describe more of the spectrum of the disease, and less on small groups of commonly co-occurring symptoms, providing a complementary analysis. Our interpretation is that, in the youngest and oldest age groups, patterns of co-occurrence of reported symptoms do change, particularly for pairs of symptoms, however, we do not observe significant changes to the overall spectrum of the disease, which can still be broadly described by number of symptoms experienced, and then the relative contribution of upper respiratory tract symptoms, or gastrointestinal symptoms. Across Pillar 2, SGSS and CIS, we consistently observe a central cluster of systemic and lower respiratory tract symptoms. Upper respiratory tract symptoms are clustered close to the systemic symptoms, but further away from the gastrointestinal symptoms. The CSS dataset is the most different, where

shortness of breath, fatigue and delirium are clustered close to gastrointestinal symptoms, but further away from the main cluster of systemic, upper respiratory tract and lower respiratory tract symptoms.

S2 Supplementary Figures

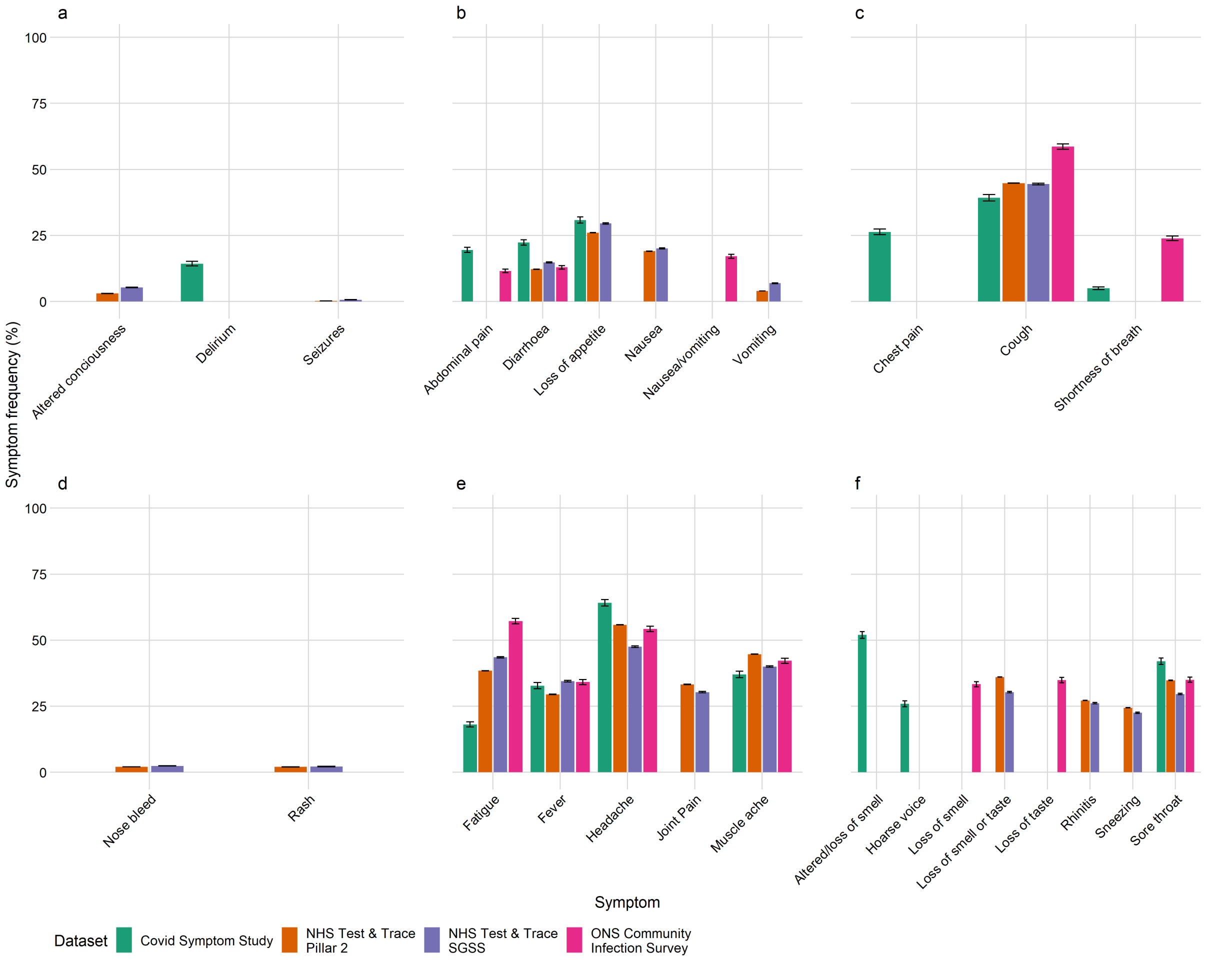

Figure S1. A plot containing the proportion of cases that develop a symptom across datasets. Each dataset records a different set of symptoms, and in some datasets multiple symptoms are considered to be one variable. Each subplot contains a different category of symptoms. **a**, altered state symptoms. **b**, gastrointestinal symptoms. **c**, lower respiratory tract symptoms. **d**, other symptoms. **e**, systemic symptoms. **f**, upper respiratory tract symptoms.

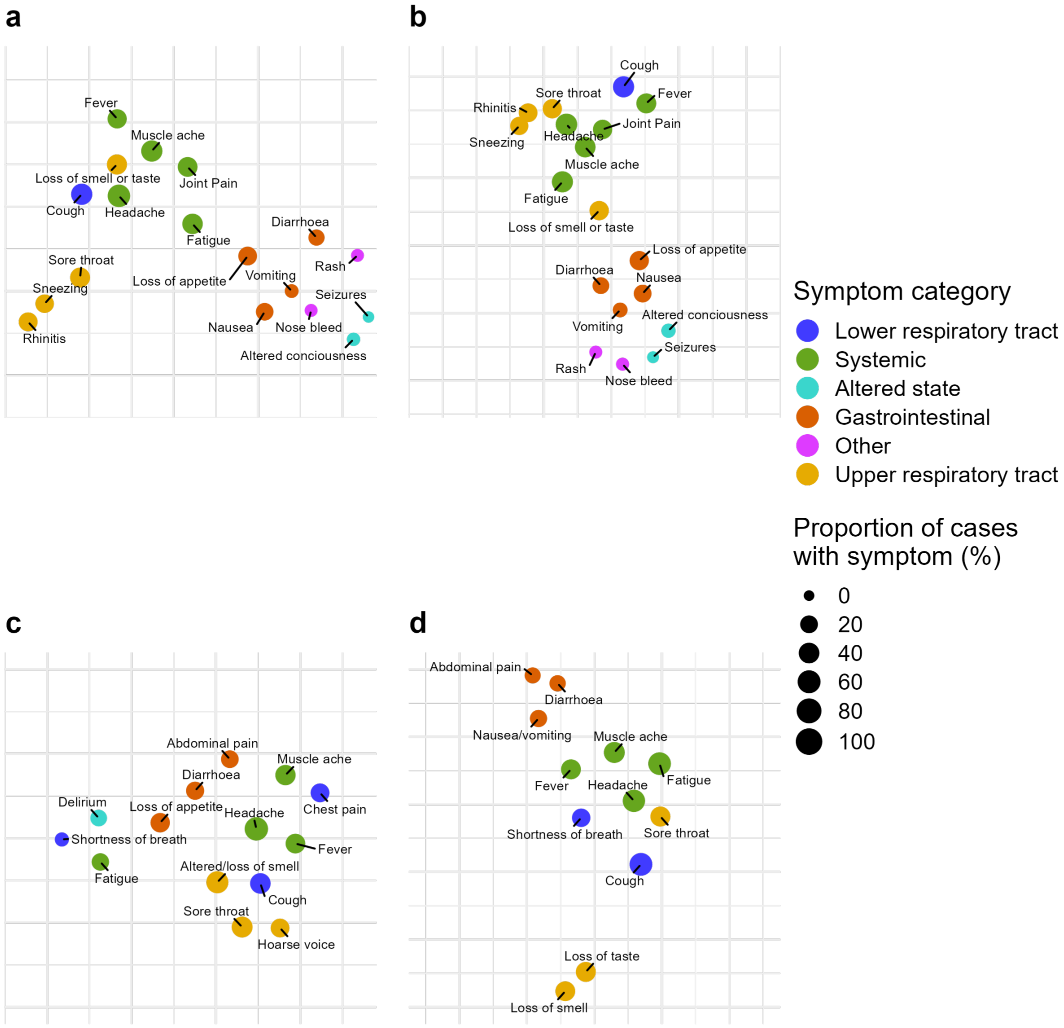

Figure S2. UMAP embeddings of SARS-CoV-2 symptoms. The algorithm attempts to place combinations of symptoms that commonly co-occur close to each other. Point size is proportional to the proportion of cases that develop a given symptom. For this embedding, the parameters were chosen to capture more of the global structure of symptoms and produces less well-defined clusters, and it was performed without any alignment between datasets. **a**. Pillar 2., **b**. SGSS, **c**. COVID Symptom Study, **d**. COVID-19 Infection Survey.

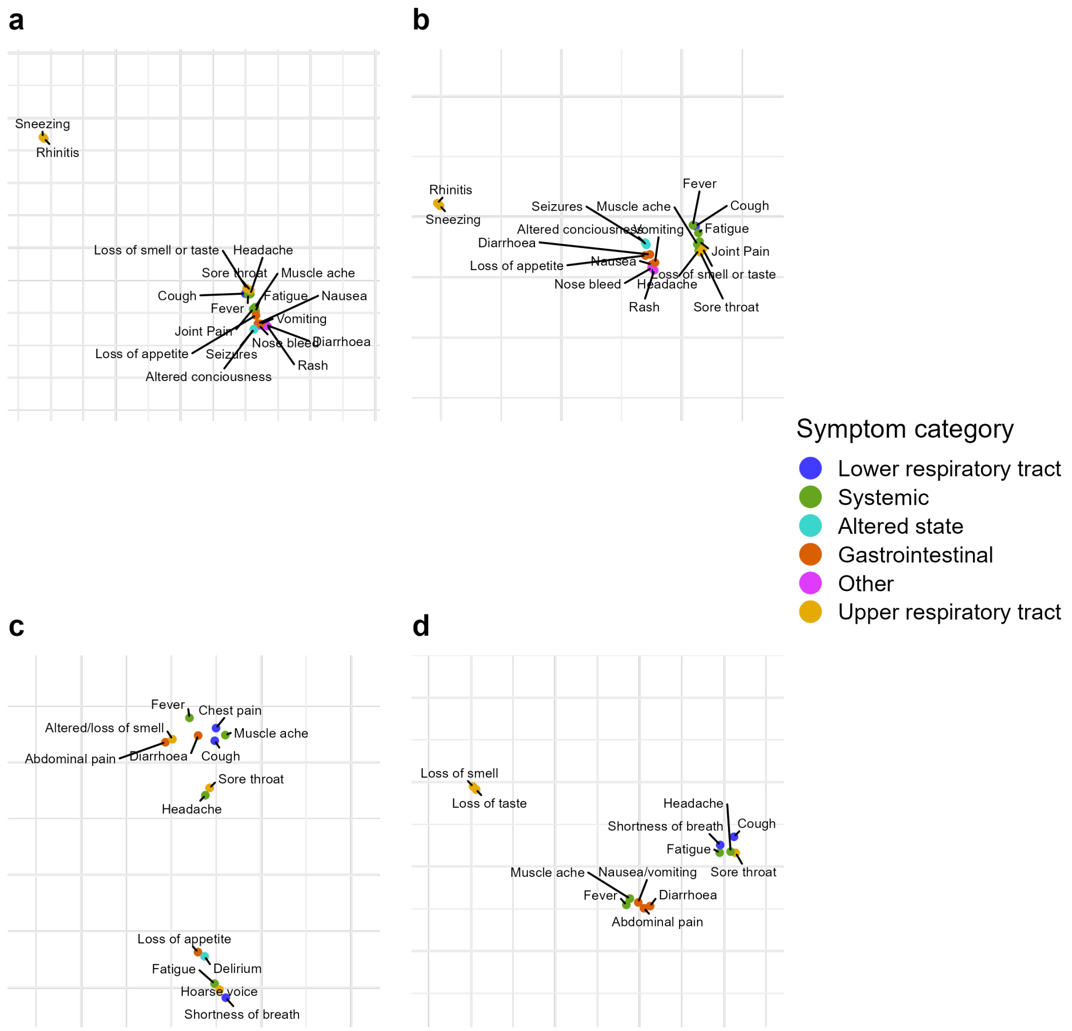

Figure S3. UMAP embeddings of SARS-CoV-2 symptoms. The algorithm attempts to place combinations of symptoms that commonly co-occur close to each other. For this embedding, the parameters were chosen to capture more of the local structure of symptoms and produces less well-defined clusters, and it was performed without any alignment between datasets. **a**. Pillar 2, **b**. SGSS, **c**. COVID Symptom Study, **d**. COVID-19 Infection Survey.

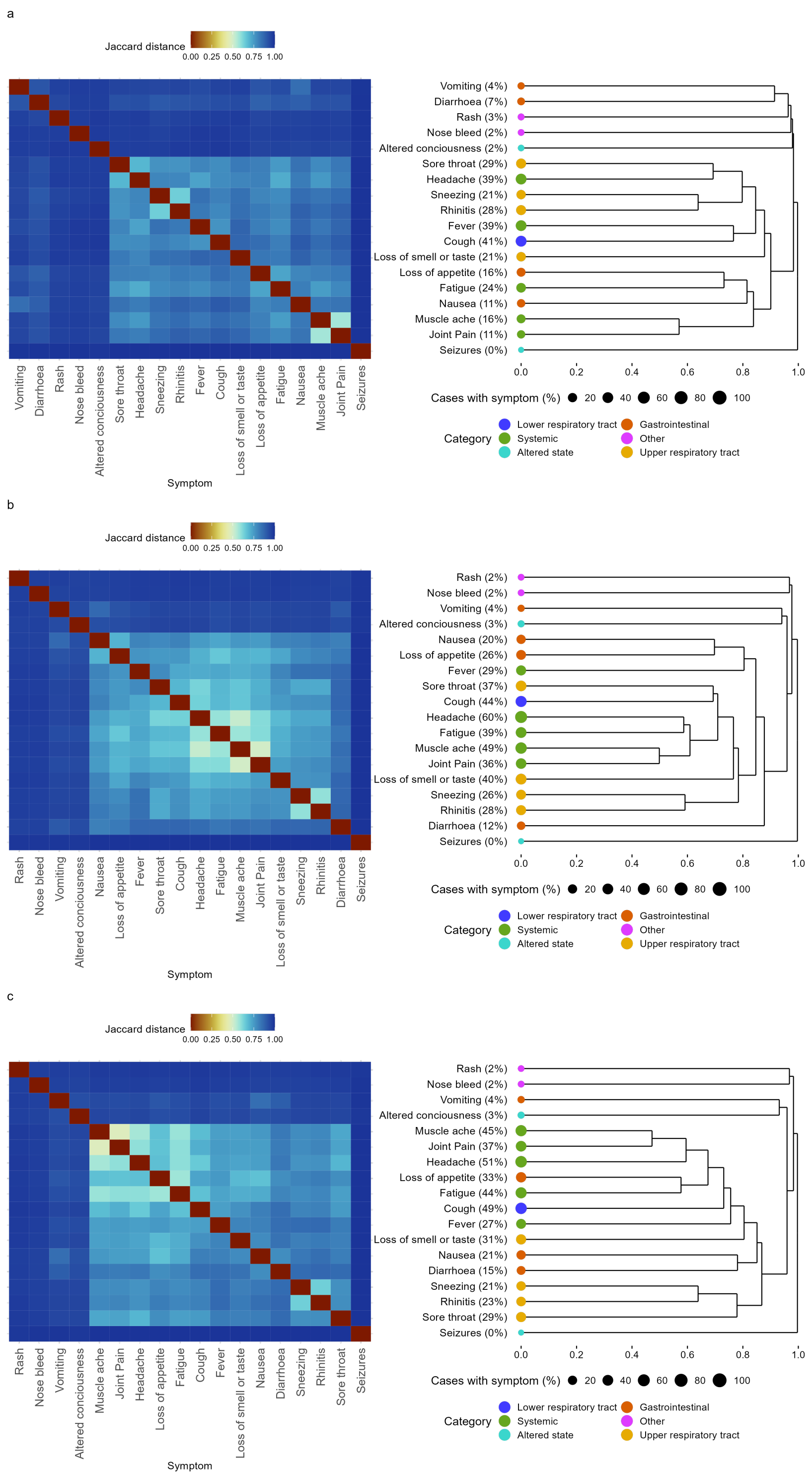

Figure S4. Hierarchical clustering of the Pillar 2 dataset with age stratification. Jaccard distance matrices between symptoms adjacent to associated dendrograms were obtained through hierarchical clustering under complete linkage. The symptom category is denoted using coloured points at the roots of the dendrogram. The central columns give the name of the symptom with the percentage of symptomatic cases who exhibit symptoms in the dataset. **a**. Children, **b**. Adults, **c**. Elders.

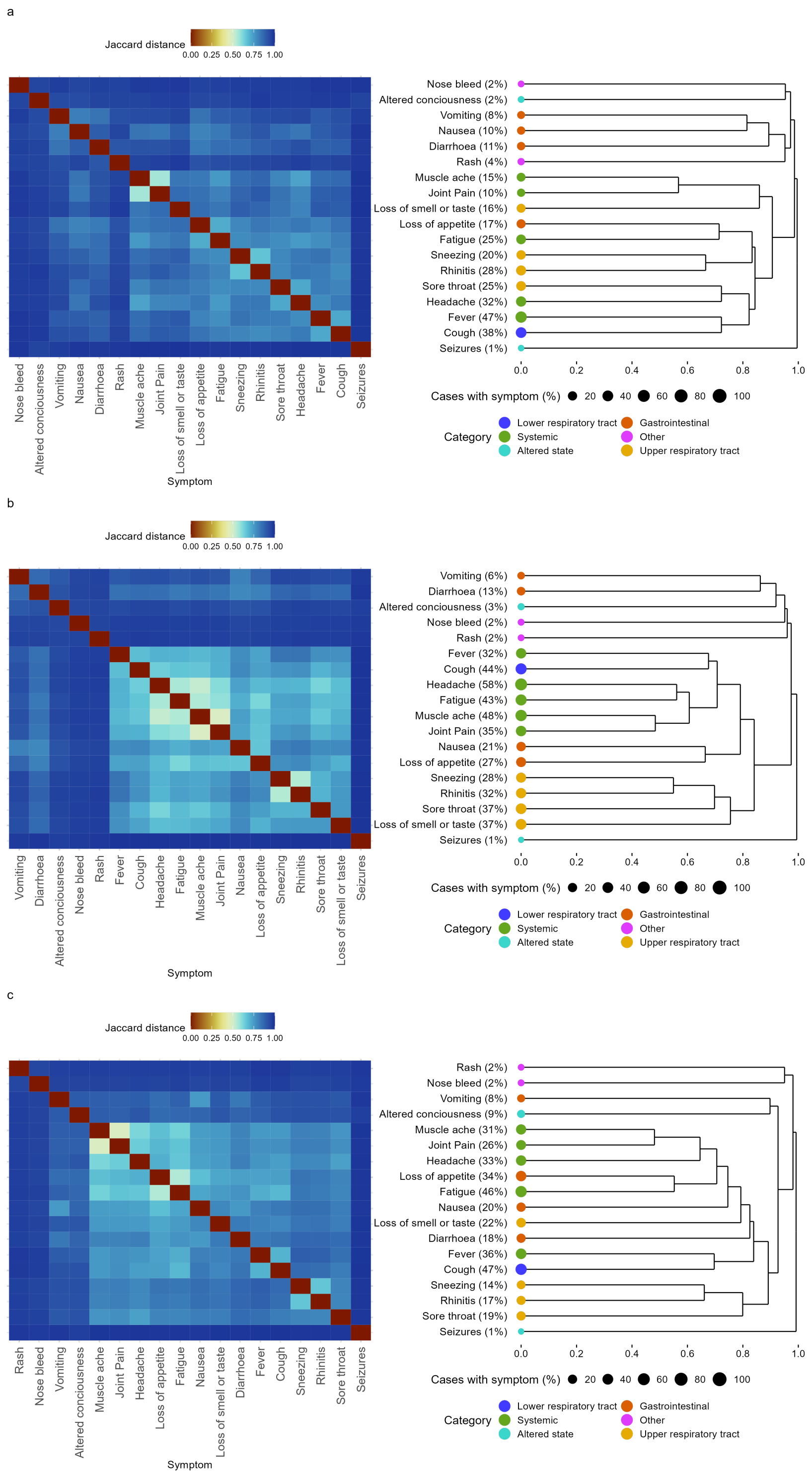

Figure S5. Hierarchical clustering of the SGSS dataset with age stratification. Jaccard distance matrices between symptoms adjacent to associated dendrograms were obtained through hierarchical clustering under complete linkage. The symptom category is denoted using coloured points at the roots of the dendrogram. The central columns give the name of the symptom with the percentage of symptomatic cases who exhibit symptoms in the dataset. **a**. Children, **b**. Adults, **c**. Elders.

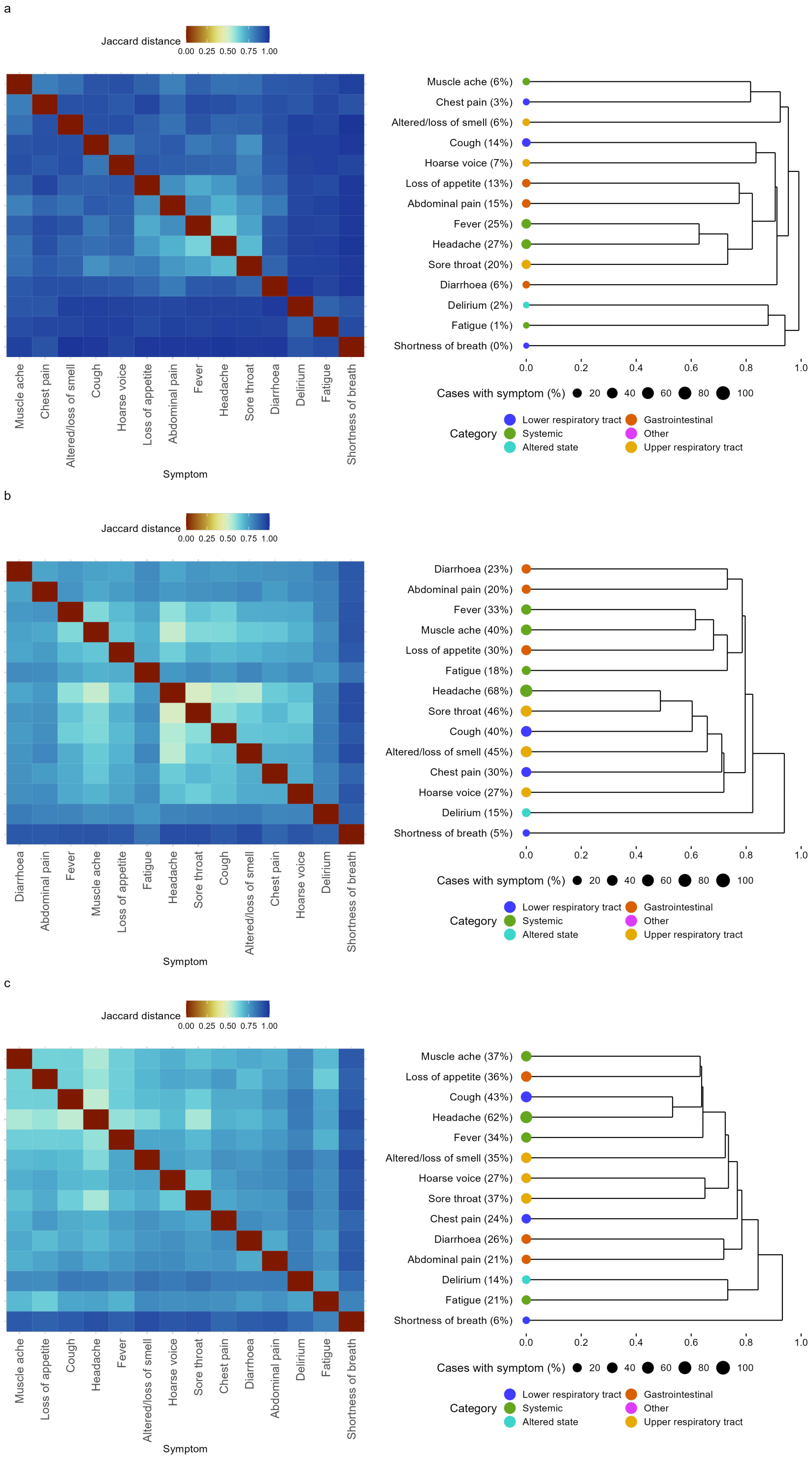

Figure S6. Hierarchical clustering of the COVID Symptom Study dataset with age stratification. Jaccard distance matrices between symptoms adjacent to associated dendrograms were obtained through hierarchical clustering under complete linkage. The symptom category is denoted using coloured points at the roots of the dendrogram. The central columns give the name of the symptom with the percentage of symptomatic cases who exhibit symptoms in the dataset. **a**. Children, **b**. Adults, **c**. Elders.

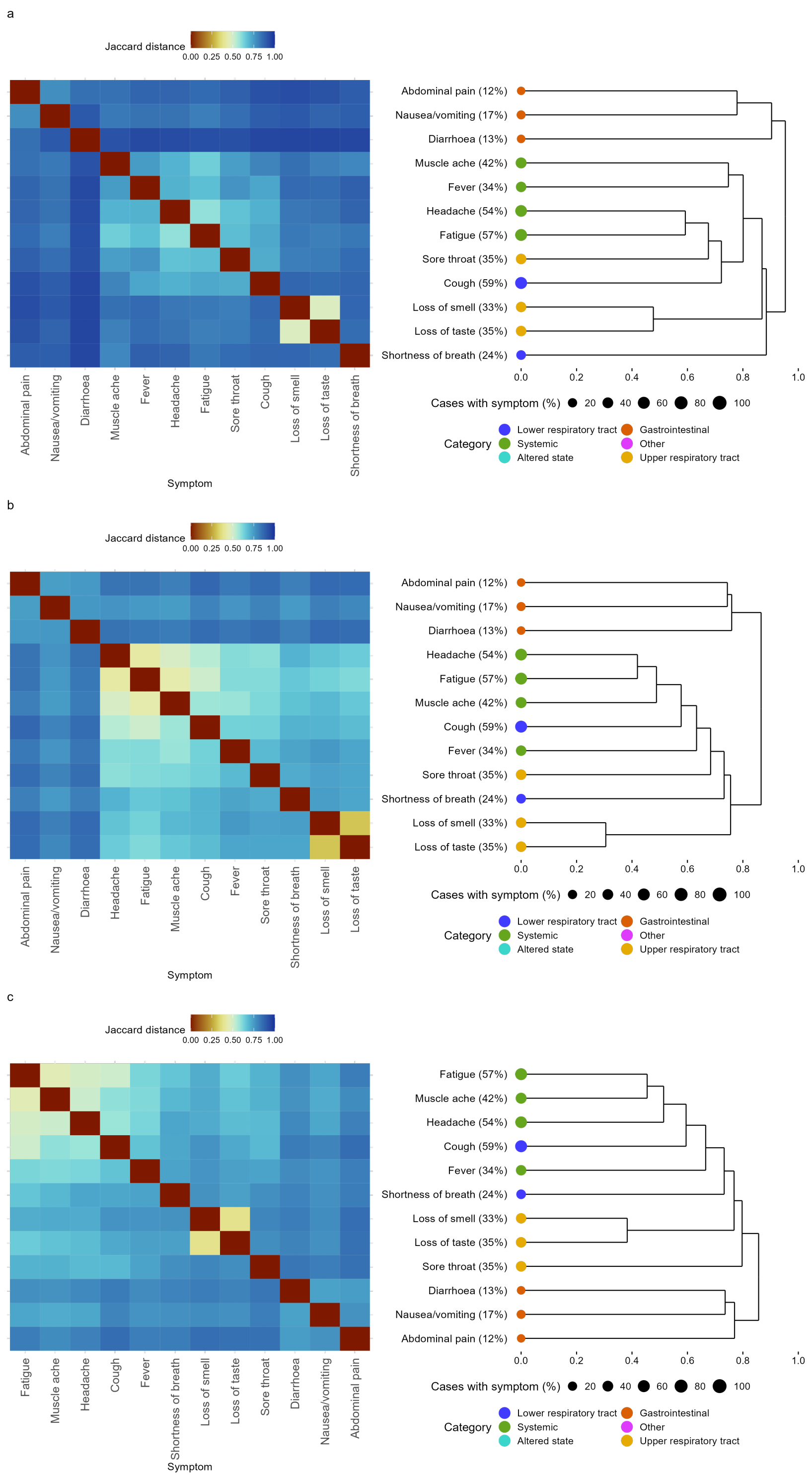

Figure S7. Hierarchical clustering of the COVID-19 Infection Survey dataset with age stratification. Jaccard distance matrices between symptoms adjacent to associated dendrograms were obtained through hierarchical clustering under complete linkage. The symptom category is denoted using coloured points at the roots of the dendrogram. The central columns give the name of the symptom with the percentage of symptomatic cases who exhibit symptoms in the dataset. **a**. Children, **b**. Adults, **c**. Elders.

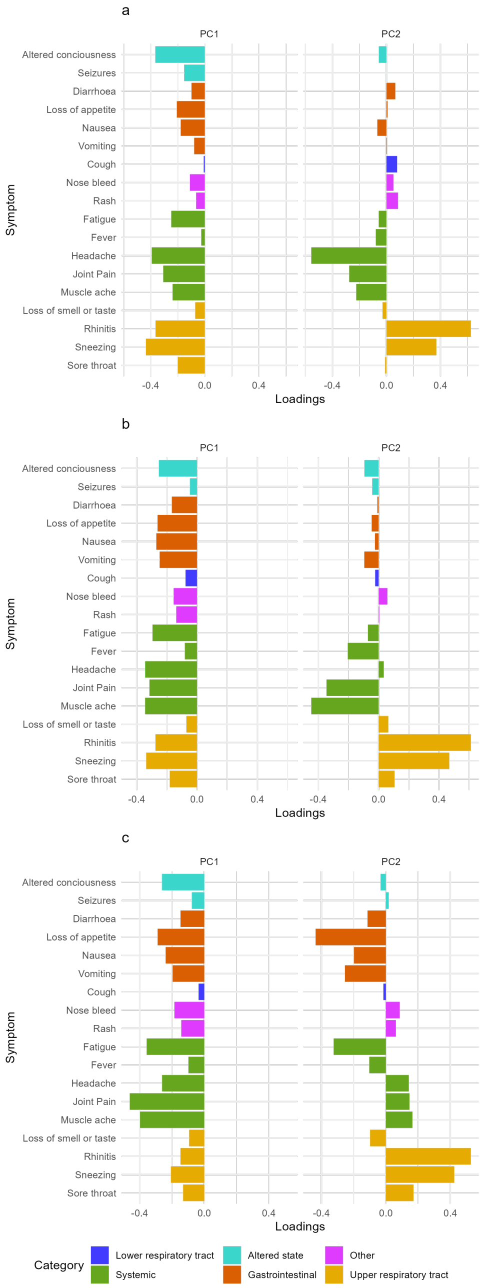

Figure S8. Logistic Principal Components Analysis (LPCA) results performed on the Pillar 2 dataset with age stratification. For each stratum, elements of the principal components are visualised as vertical bar plots. Each vector is insensitive to overall multiplication by −1. Symptom categories are labelled by colours. **a**. Children, **b**. Adults, **c**. Elders.

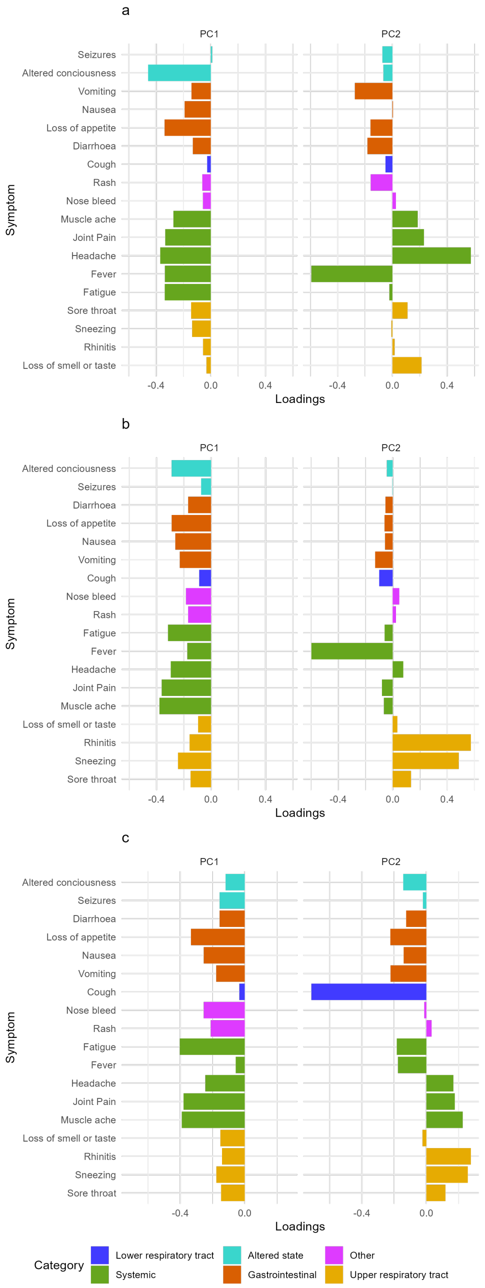

Figure S9. Logistic Principal Components Analysis (LPCA) results performed on the SGSS dataset with age stratification. For each stratum, elements of the principal components are visualised as vertical bar plots. Each vector is insensitive to overall multiplication by -1. Symptom categories are labelled by colours. **a**. Children, **b**. Adults, **c**. Elders.

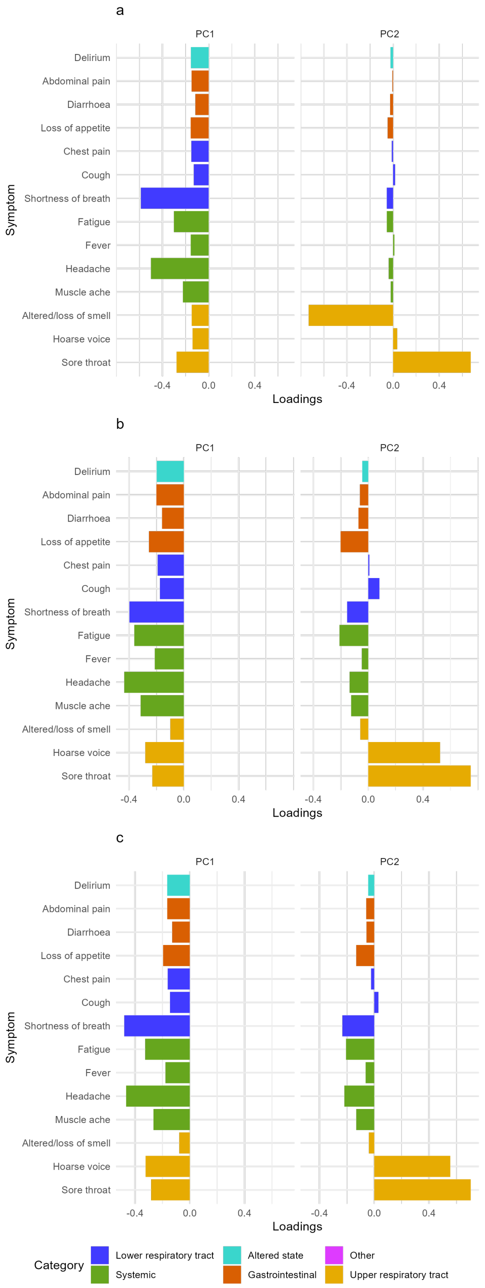

Figure S10. Logistic Principal Components Analysis (LPCA) results performed on the COVID Symptom Study dataset with age stratification. For each stratum, elements of the principal components are visualised as vertical bar plots. Each vector is insensitive to overall multiplication by -1. Symptom categories are labelled by colours. **a**. Children, **b**. Adults, **c**. Elders.

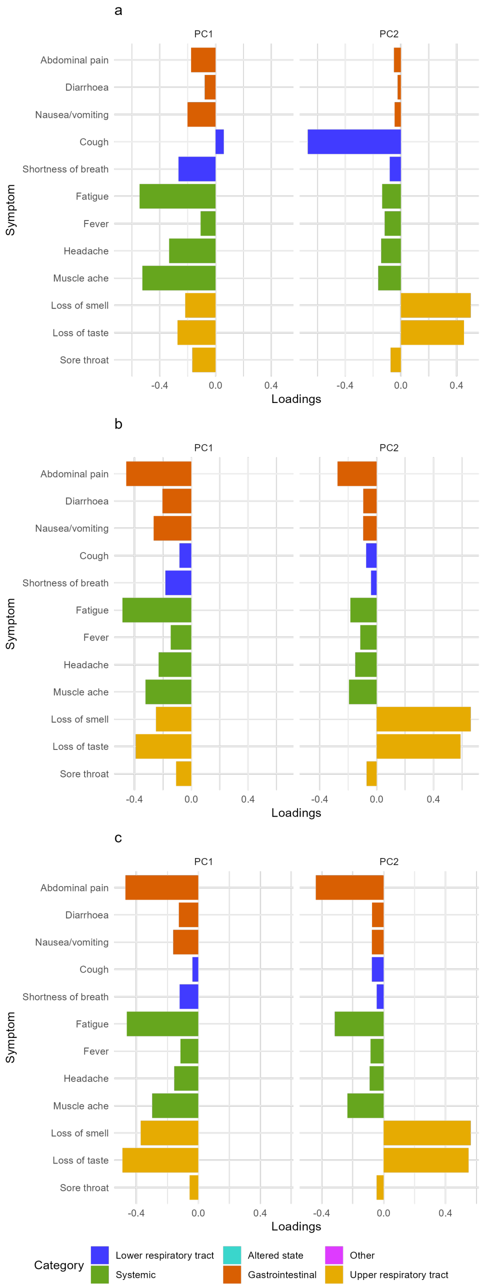

Figure S11. Logistic Principal Components Analysis (LPCA) results performed on the COVID-19 Infection Survey dataset with age stratification. For each stratum, elements of the principal components are visualised as vertical bar plots. Each vector is insensitive to overall multiplication by -1. Symptom categories are labelled by colours. **a**. Children, **b**. Adults, **c**. Elders.

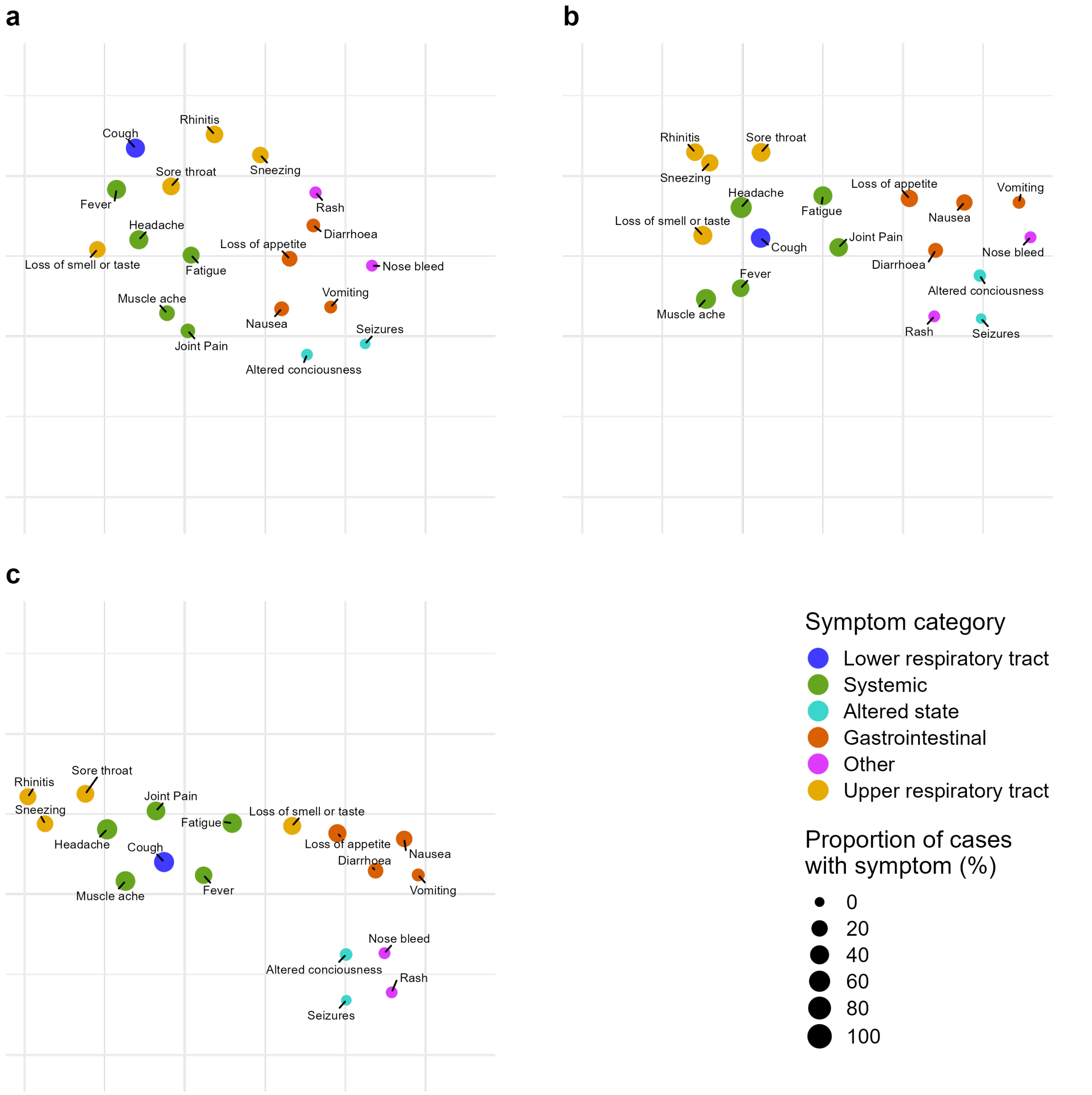

Figure S12. UMAP embeddings of SARS-CoV-2 symptoms performed on Pillar 2 dataset with age stratification. The algorithm attempts to place combinations of symptoms that commonly co-occur close to each other. Point size is proportional to the proportion of cases that develop a given symptom. For this embedding, the parameters were chosen to capture more of the global structure of symptoms and produces less well-defined clusters. **a**. Children, **b**. Adults, **c**. Elders.

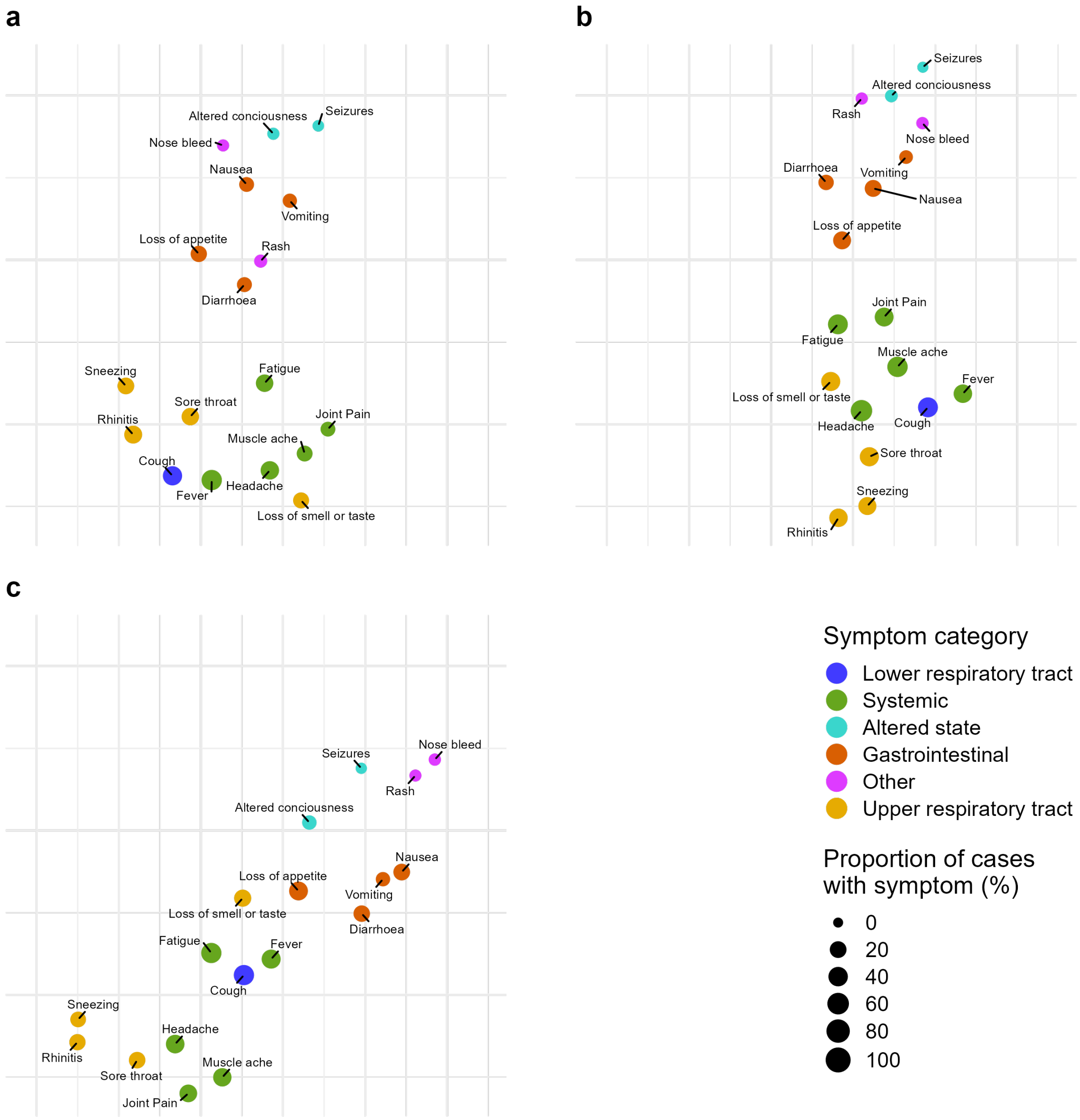

Figure S13. UMAP embeddings of SARS-CoV-2 symptoms performed on SGSS dataset with age stratification. The algorithm attempts to place combinations of symptoms that commonly co-occur close to each other. Point size is proportional to the proportion of cases that develop a given symptom. For this embedding, the parameters were chosen to capture more of the global structure of symptoms and produces less well-defined clusters. **a**. Children, **b**. Adults, **c**. Elders.

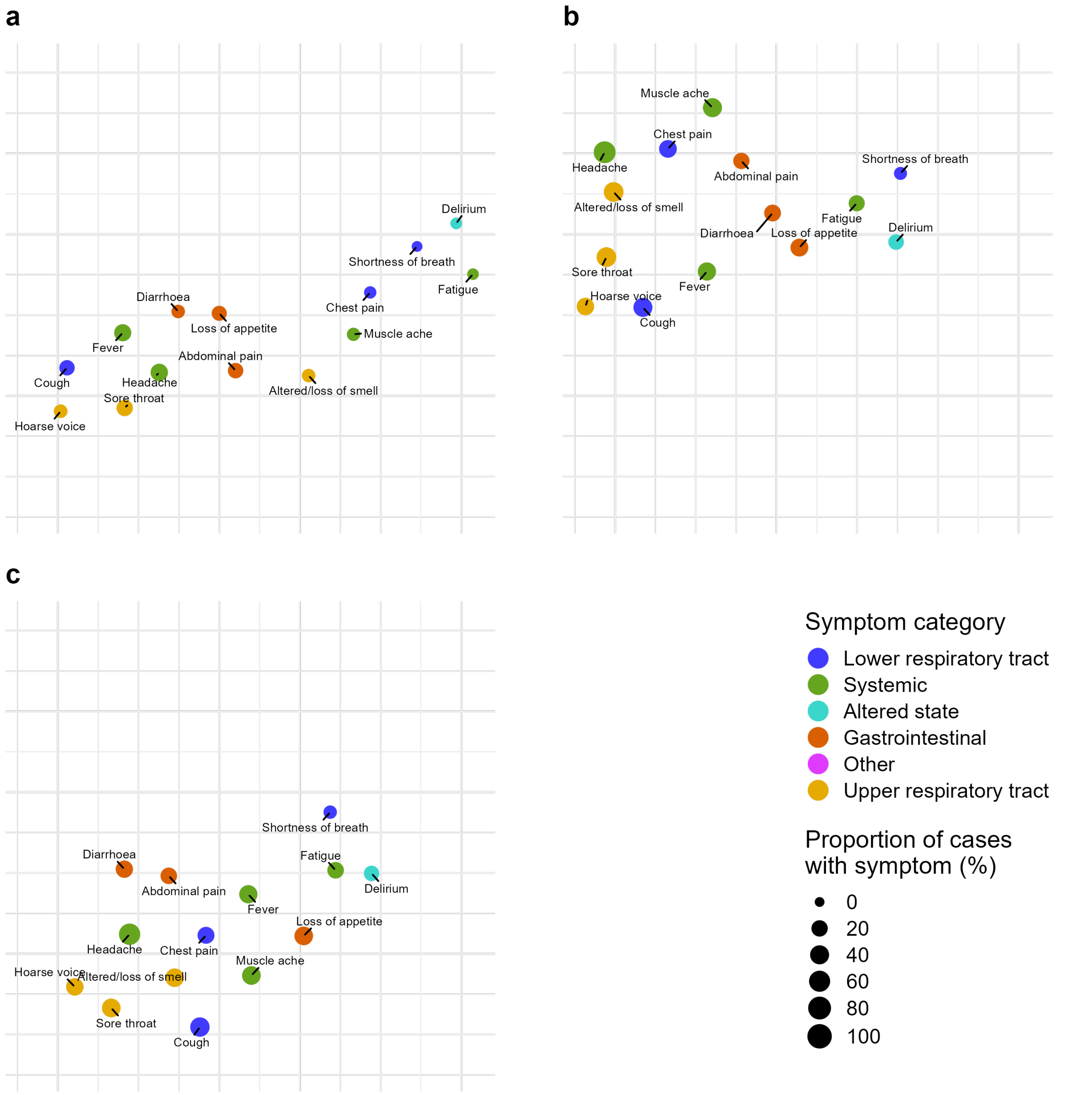

Figure S14. UMAP embeddings of SARS-CoV-2 symptoms performed on COVID Symptom Study dataset with age stratification. The algorithm attempts to place combinations of symptoms that commonly co-occur close to each other. Point size is proportional to the proportion of cases that develop a given symptom. For this embedding, the parameters were chosen to capture more of the global structure of symptoms and produces less well-defined clusters. **a**. Children, **b**. Adults, **c**. Elders.

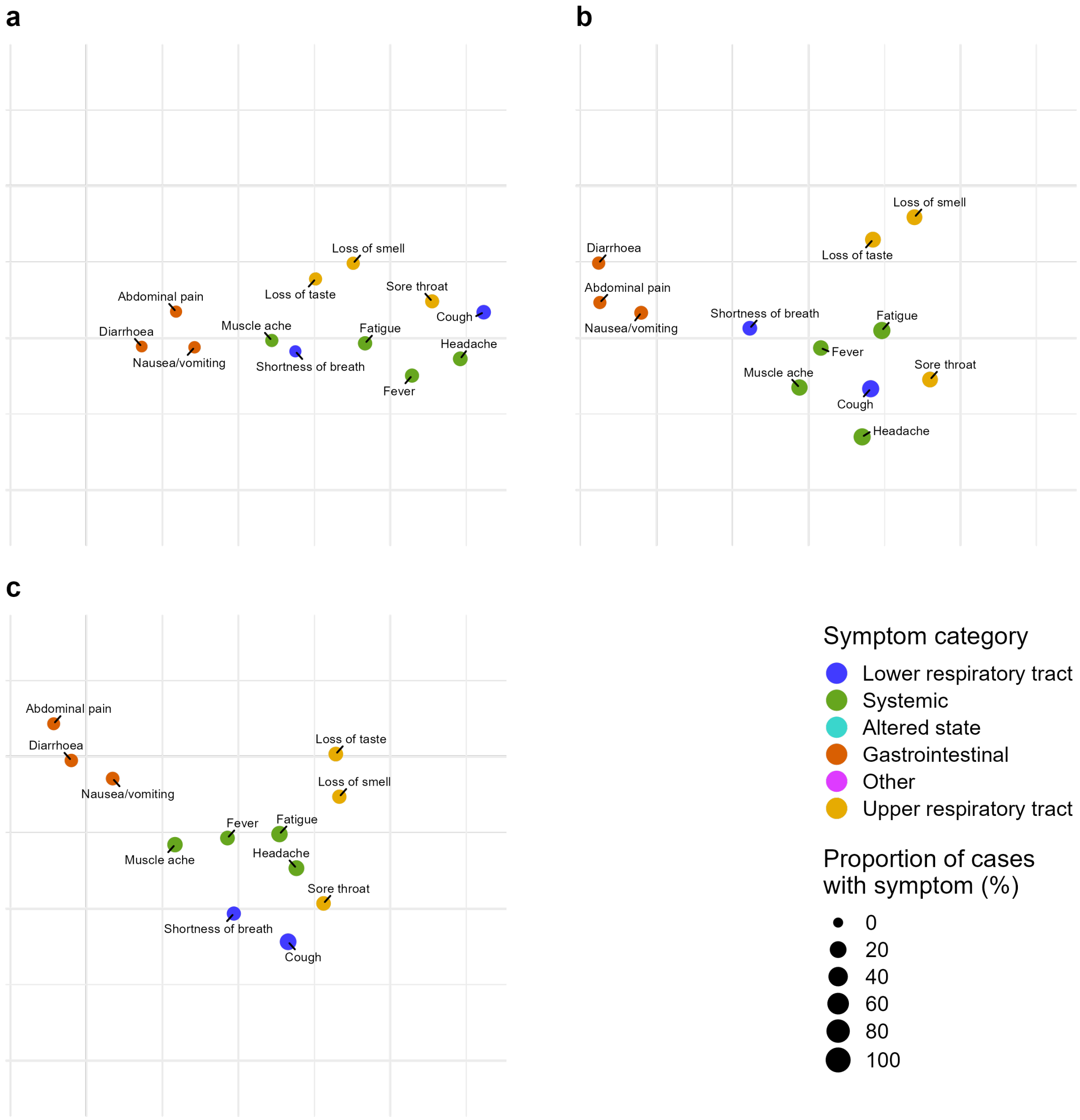

Figure S15. UMAP embeddings of SARS-CoV-2 symptoms performed on COVID-19 Infection Survey dataset with age stratification. The algorithm attempts to place combinations of symptoms that commonly co-occur close to each other. Point size is proportional to the proportion of cases that develop a given symptom. For this embedding, the parameters were chosen to capture more of the global structure of symptoms and produces less well-defined clusters. **a**. Children, **b**. Adults, **c**. Elders.

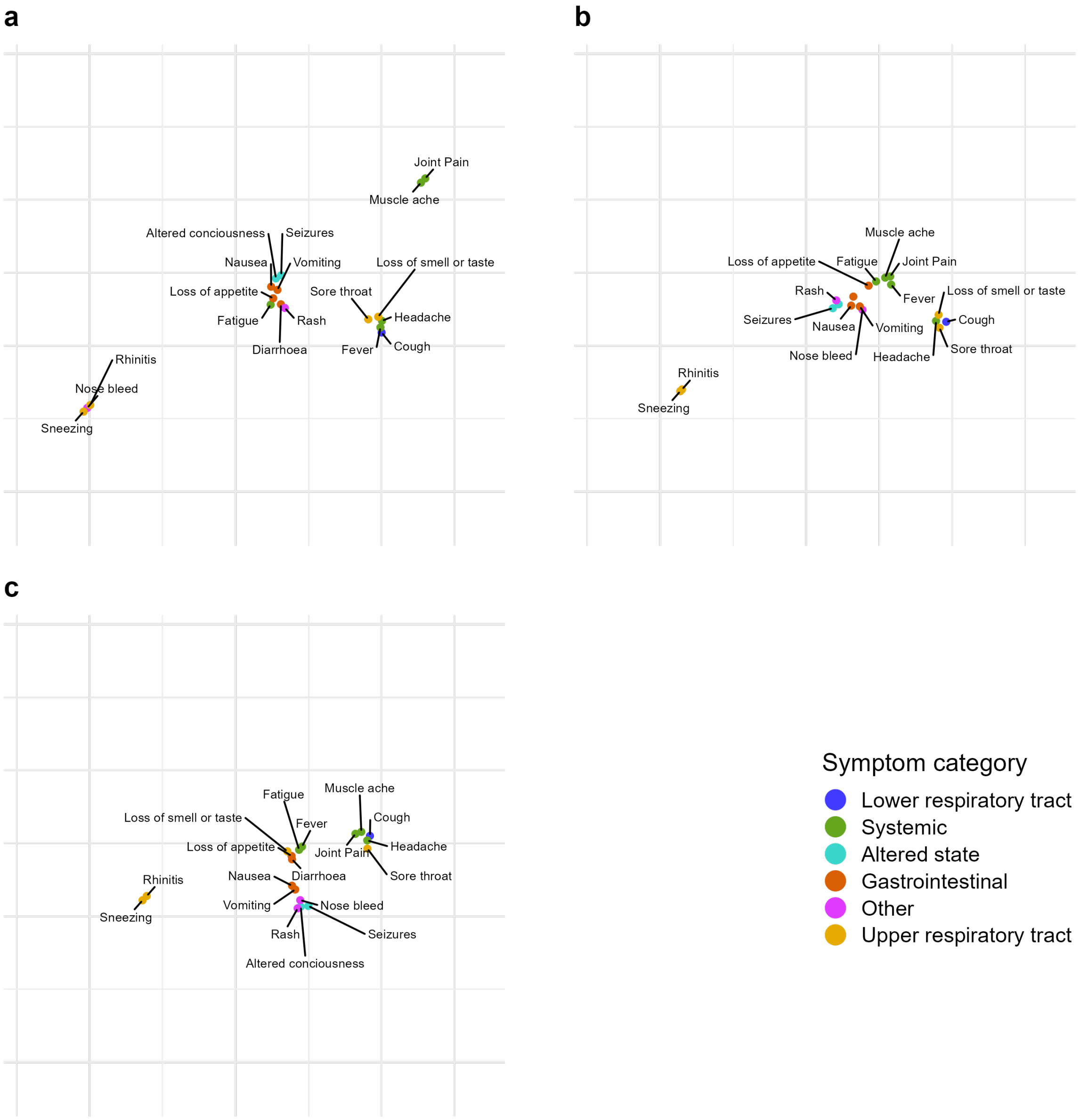

Figure S16. UMAP embeddings of SARS-CoV-2 symptoms performed on Pillar 2 dataset with age stratification. The algorithm attempts to place combinations of symptoms that commonly co-occur close to each other. For this embedding, the parameters were chosen to produce well-separated symptom clusters. **a**. Children, **b**. Adults, **c**. Elders.

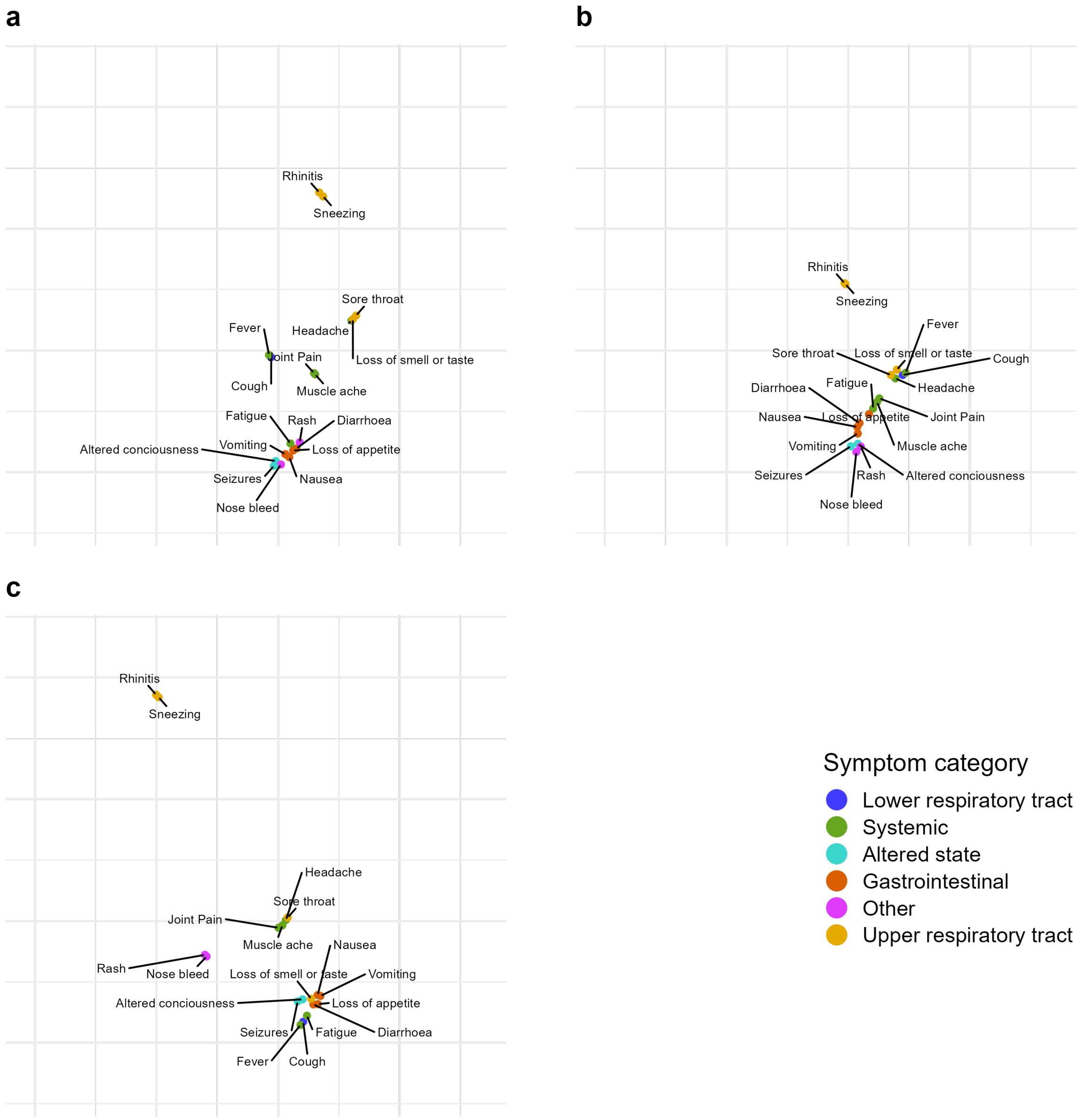

Figure S17. UMAP embeddings of SARS-CoV-2 symptoms performed on SGSS dataset with age stratification. The algorithm attempts to place combinations of symptoms that commonly co-occur close to each other. For this embedding, the parameters were chosen to produce well-separated symptom clusters. **a**. Children, **b**. Adults, **c**. Elders.

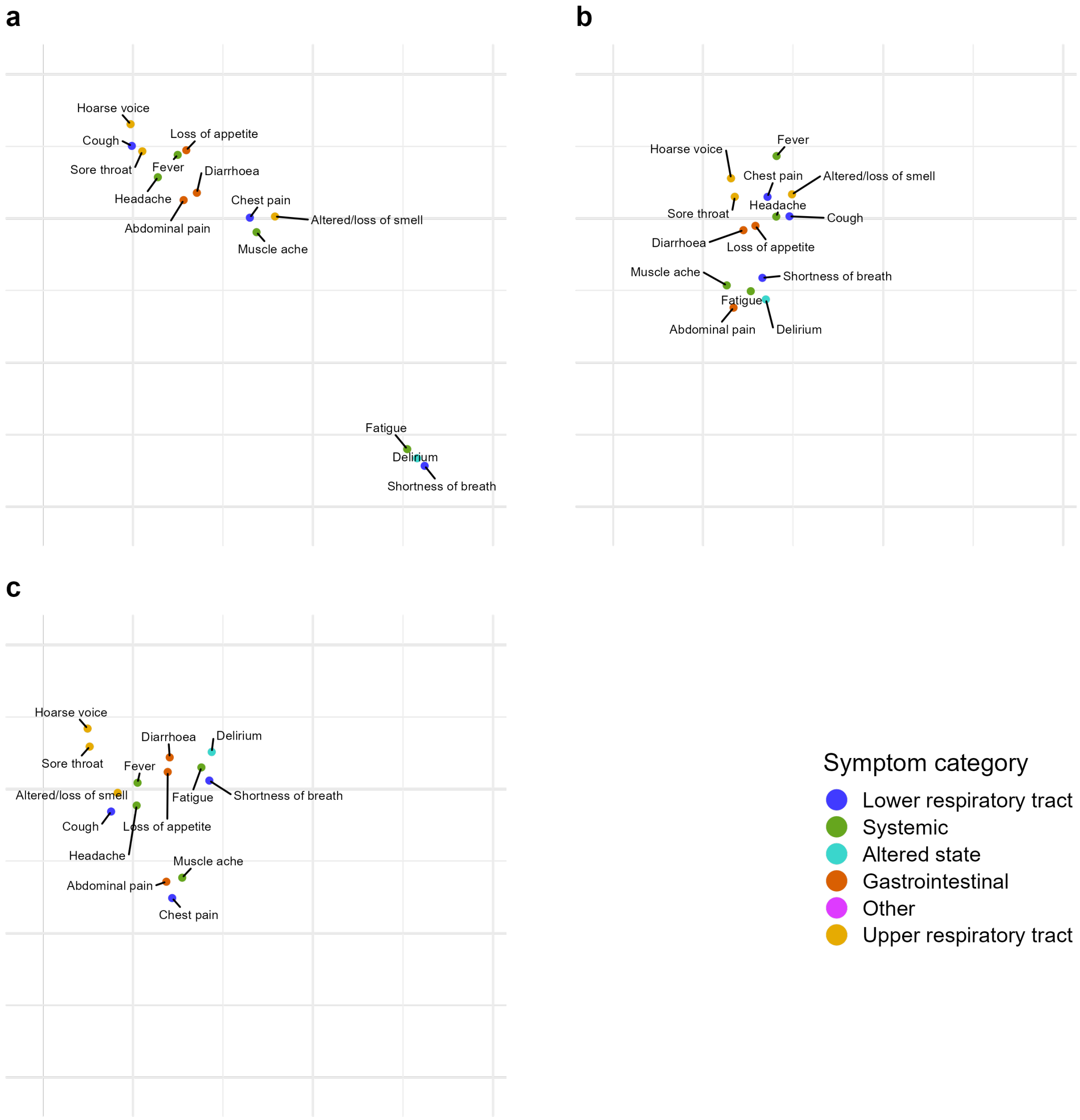

Figure S18. UMAP embeddings of SARS-CoV-2 symptoms performed on COVID Symptom Study dataset with age stratification. The algorithm attempts to place combinations of symptoms that commonly co-occur close to each other. For this embedding, the parameters were chosen to produce well-separated symptom clusters. **a**. Children, **b**. Adults, **c**. Elders.

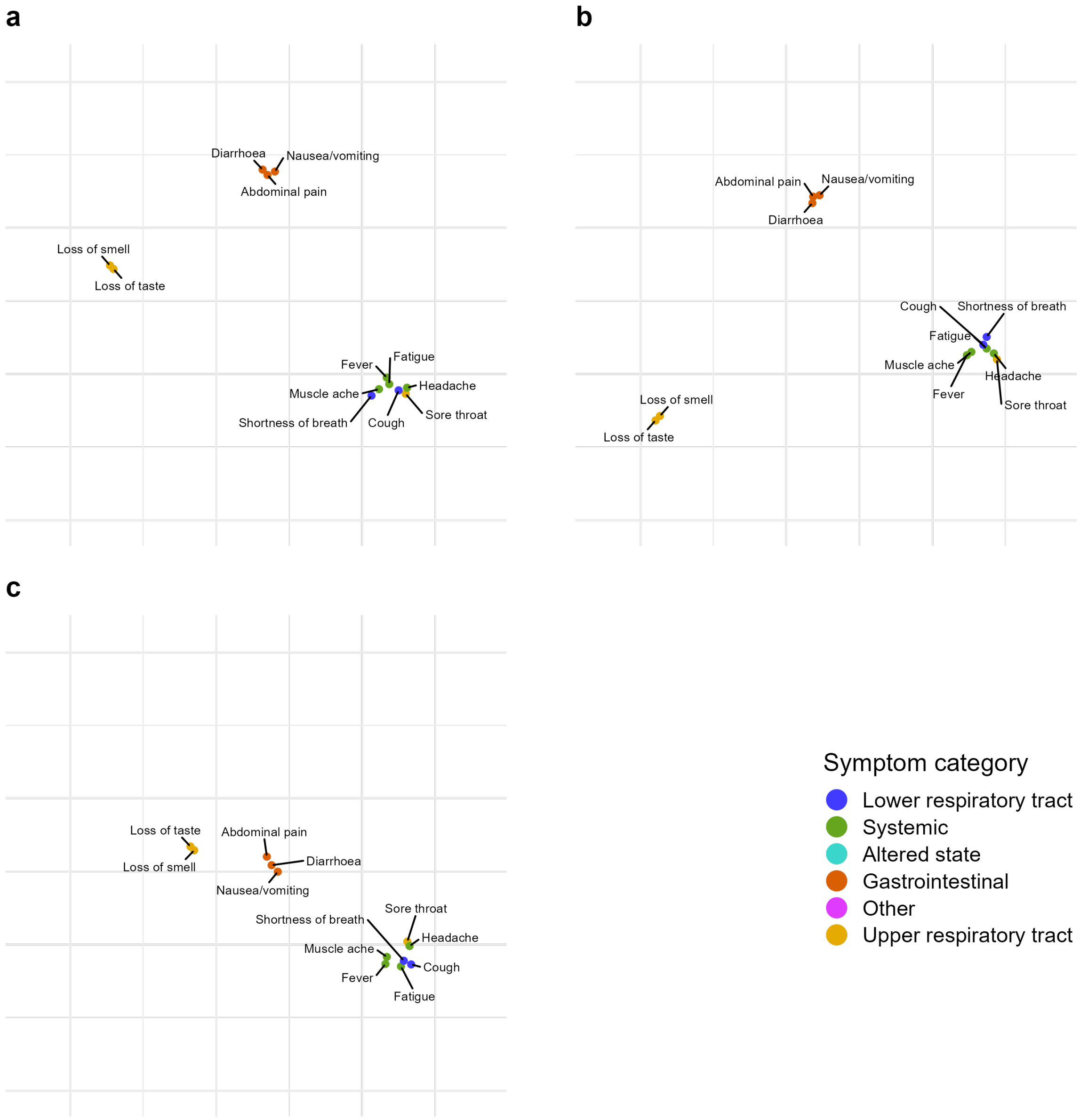

Figure S19. UMAP embeddings of SARS-CoV-2 symptoms performed on COVID-19 Infection Survey dataset with age stratification. The algorithm attempts to place combinations of symptoms that commonly co-occur close to each other. For this embedding, the parameters were chosen to produce well-separated symptom clusters. **a**. Children, **b**. Adults, **c**. Elders.

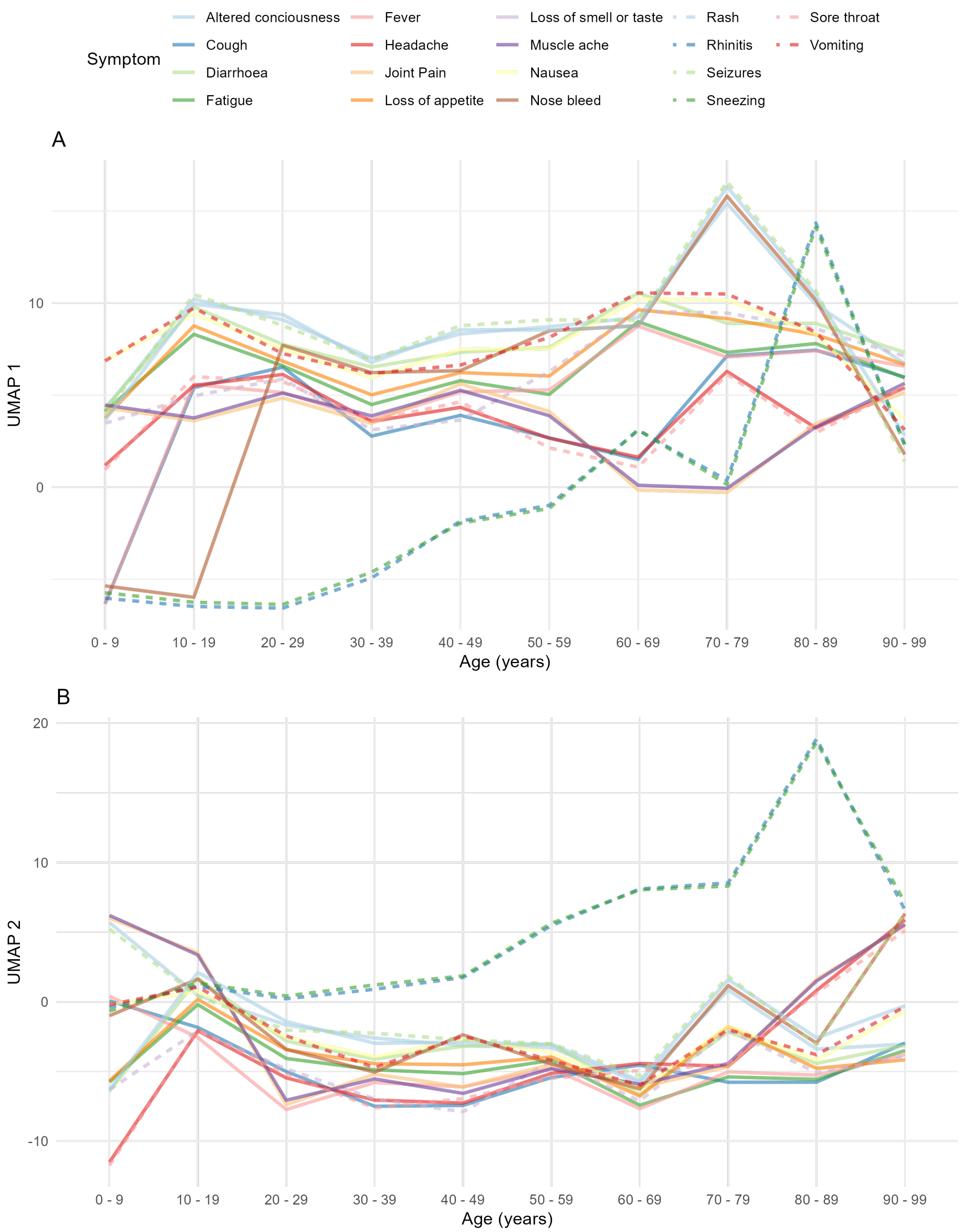

Figure S20. Marginal plots of AlignedUMAP embeddings of SARS-CoV-2, for Pillar 2 data age-stratified into strata of length 10 years. For each strata, an optimal two- dimensional embedding into Euclidean space, denoted via UMAP 1 and UMAP 2, of the symptoms is found, subject to the loose constraint that each symptom is placed in a similar location in adjacent embeddings.

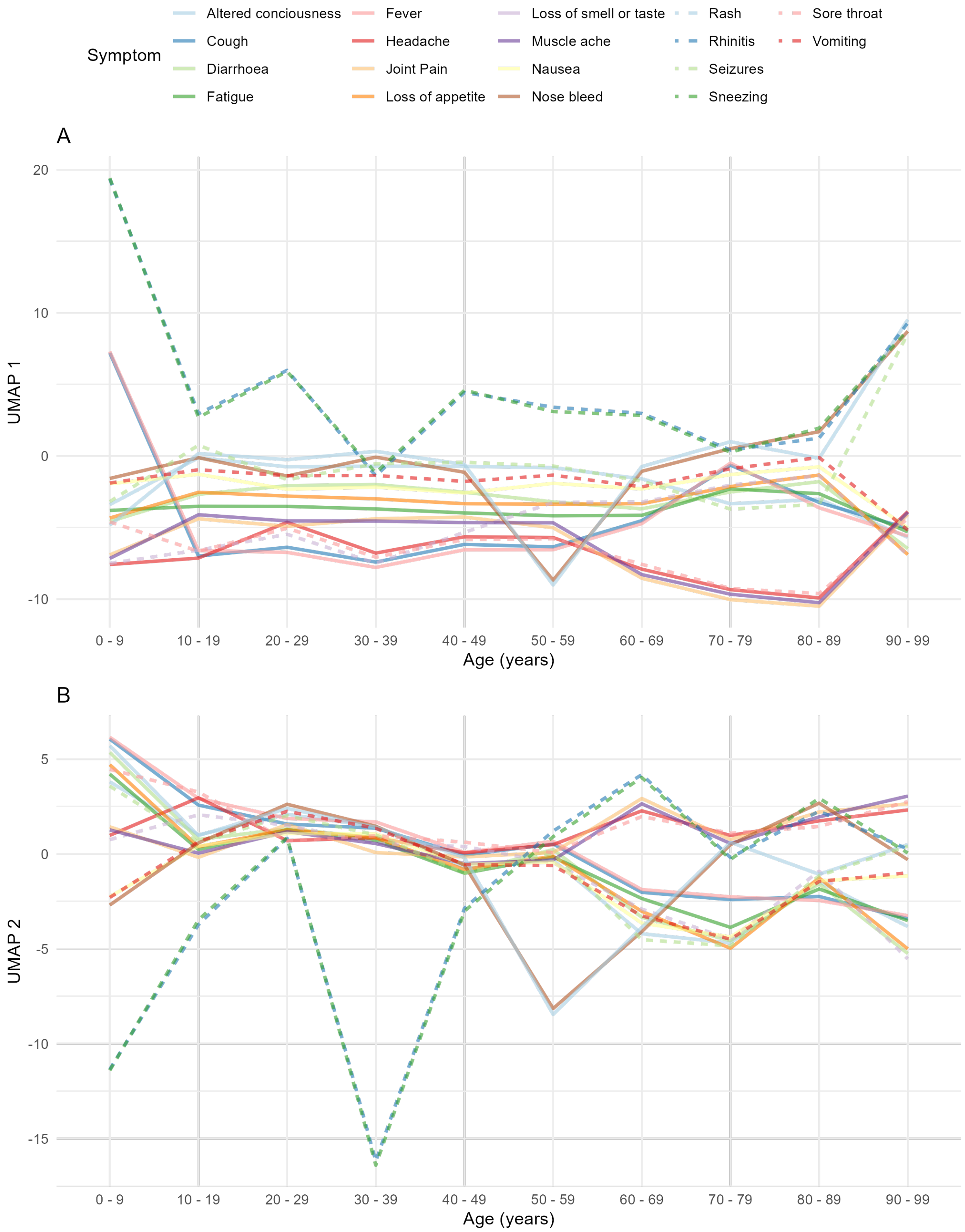

Figure S21. Marginal plots of AlignedUMAP embeddings of SARS-CoV-2, for SGSS data age-stratified into strata of length 10 years. For each strata, an optimal two- dimensional embedding into Euclidean space, denoted via UMAP 1 and UMAP 2, of the symptoms is found, subject to the loose constraint that each symptom is placed in a similar location in adjacent embeddings.

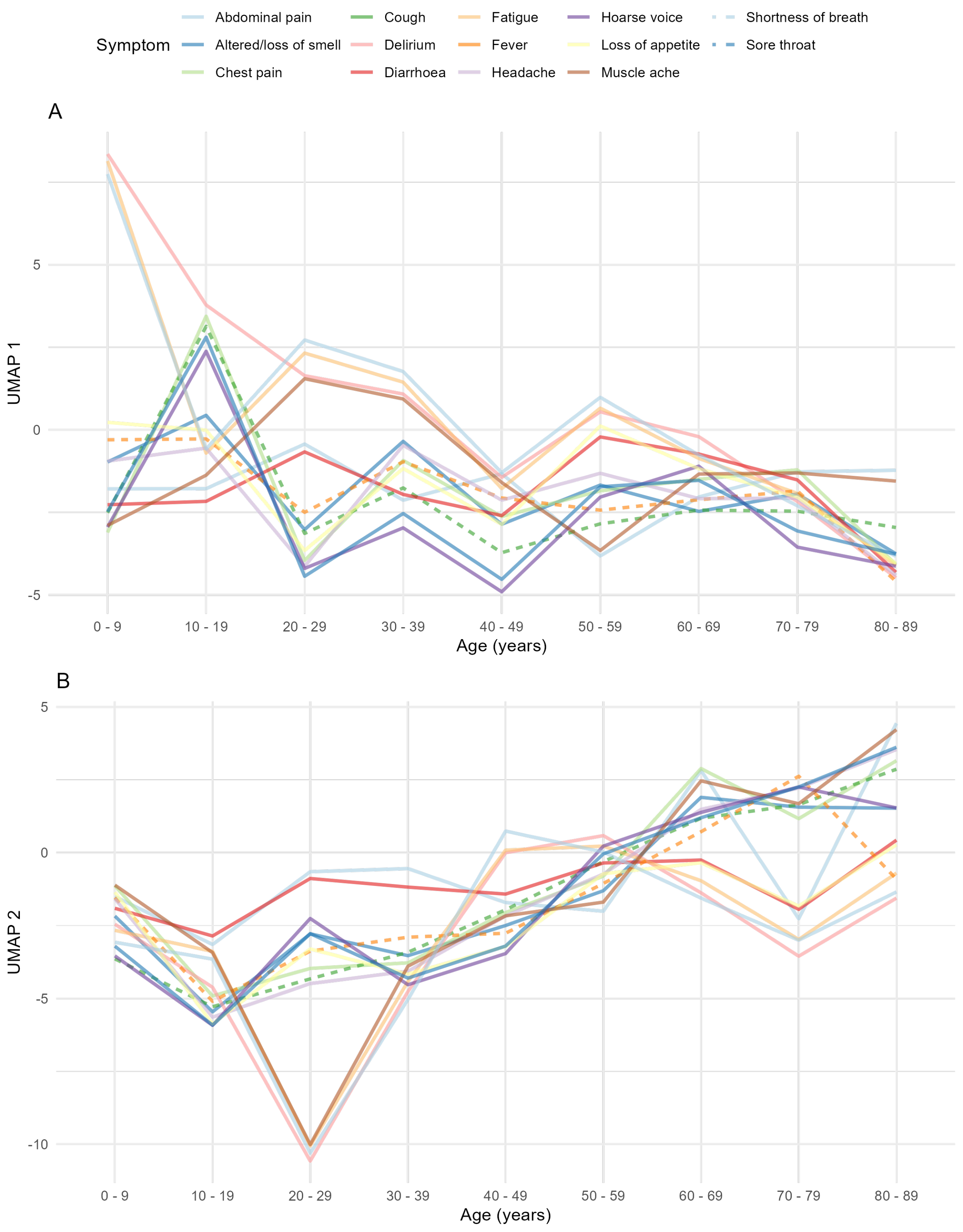

Figure S22. Marginal plots of AlignedUMAP embeddings of SARS-CoV-2, for COVID Symptom Study data age-stratified into strata of length 10 years. For each strata, an optimal two-dimensional embedding into Euclidean space, denoted via UMAP 1 and UMAP 2, of the symptoms is found, subject to the loose constraint that each symptom is placed in a similar location in adjacent embeddings.

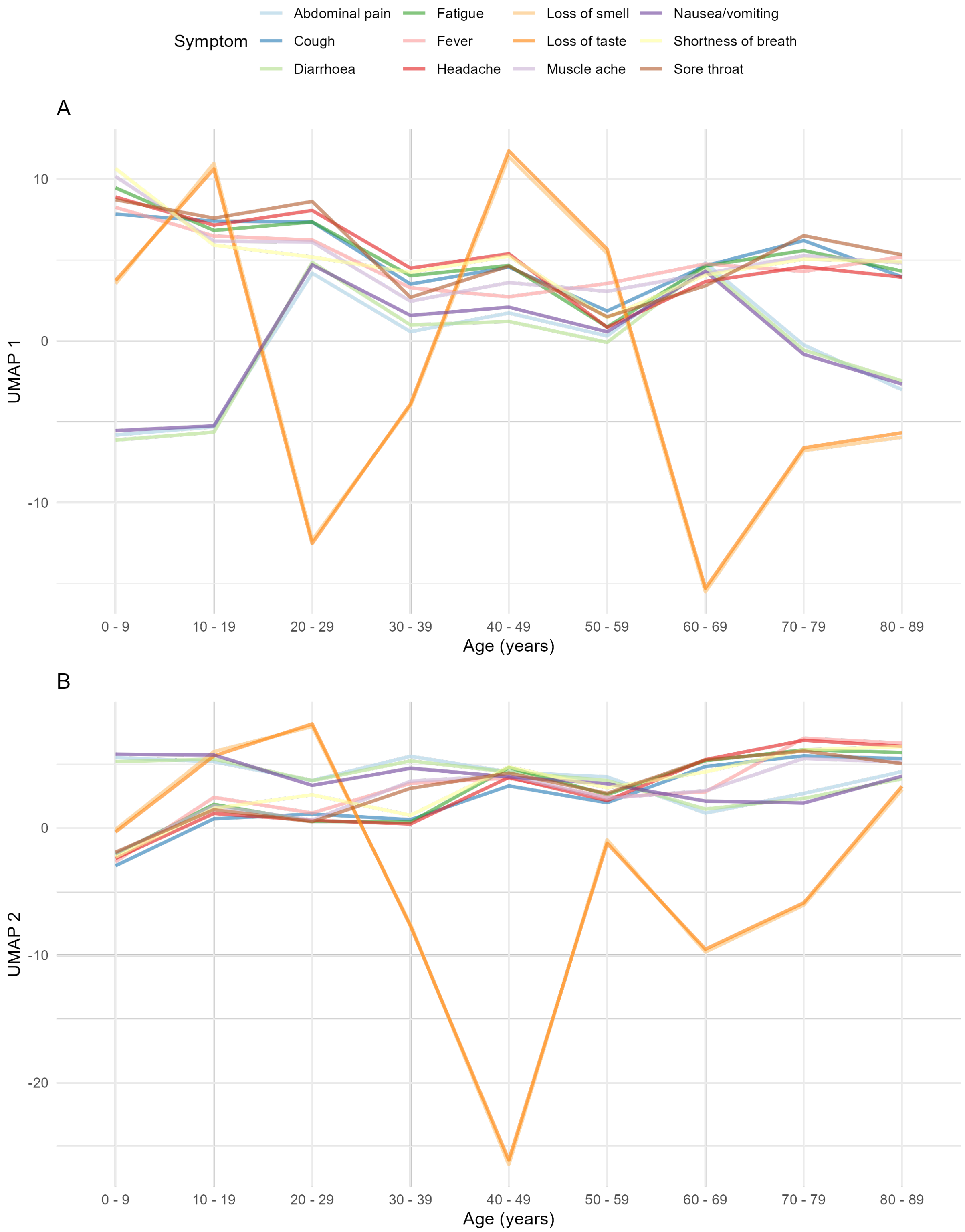

Figure S23. Marginal plots of AlignedUMAP embeddings of SARS-CoV-2, for COVID-19 Infection Survey data age-stratified into strata of length 10 years. For each strata, an optimal two- dimensional embedding into Euclidean space, denoted via UMAP 1 and UMAP 2, of the symptoms is found, subject to the loose constraint that each symptom is placed in a similar location in adjacent embeddings.
